# Beyond black and white: dissecting the genetic basis of skin depigmentation in Nellore cattle

**DOI:** 10.1007/s00335-025-10153-9

**Published:** 2025-08-04

**Authors:** Milena A. F. Campos, Hinayah Rojas de Oliveira, Gregorio M. F. de Camargo, Henrique A. Mulim, Diercles Francisco Cardoso, Raphael Bermal Costa

**Affiliations:** 1 Escola de Medicina Veterinária E Zootecnia, Universidade Federal da Bahia, Salvador, Bahia Brazil; 2 Department of Animal Sciences, Purdue University, West Lafayette, IN USA; 3 Gensys® Associated Consultants, Porto Alegre, Rio Grande Do Sul Brazil

**Keywords:** Beef cattle, Binary traits, Candidate gene, Enrichment analysis, Conditional GWAS, Melanogenesis, SNP

## Abstract

Depigmentation defects in cattle are characterized by the absence of pigment in specific skin regions, increasing susceptibility to health issues and often leading to early culling. In Nellore cattle, depigmentation is primarily observed at the tail tip, mucous membranes, and as small patches across the body. This study aimed to estimate genetic parameters and perform a genome-wide association study (GWAS) for depigmentation in Nellore cattle. Data were sourced from the DeltaGen® breeding program, provided by Gensys®. Phenotypic records included 182,964 Nellore cattle, with a 6.8% incidence of depigmentation. Of these, 28,655 genotyped animals and 385,079 SNPs were available for the analysis. The ultra-fast generalized linear mixed model for binary traits (fastGWA-GLMM) was used for the GWAS, while variance components were estimated using a Bayesian threshold model and single-step methodology. The heritability of depigmentation was estimated at 0.12 on the observed scale and 0.54 on the liability scale. The GWAS identified 1,011 significant SNPs (p < 0.05 after Bonferroni correction) associated with depigmentation defects, located across chromosomes BTA6, BTA12, and BTA22. However, after performing a conditional GWAS to account for the top signal on BTA22, the original signal in the *MITF* region was no longer detected. In total, 234 genes were identified near the associated SNPs, including 129 protein-coding genes. Functional enrichment highlighted *MITF*, *KIT* and *EDNRB* as biologically relevant candidate genes. The gene ontology analysis highlighted biological processes related to melanogenesis, pigmentation, and hypopigmentation phenotypes, while the QTL enrichment analysis identified significant associations on BTA6 and BTA22. These findings improve our understanding of the genetic basis of depigmentation in Nellore cattle and may contribute to future selection strategies.

**Supplementary Information:**

The online version contains supplementary material available at 10.1007/s00335-025-10153-9.

## Introduction

Nellore is the predominant beef cattle breed raised in Brazil and plays a significant role in the country's livestock production due to its adaptability to extensive pasture-based systems in tropical environments (Nunes et al. 2024). Nellore cattle are predominantly raised outdoors, grazing on natural and cultivated pastures year-round, which exposes them to high levels of solar radiation and variable climatic conditions. A notable feature of the breed is its short, dense white coat, which helps to reflect solar radiation and reduce heat absorption, supporting thermoregulation (Costa et al. 2018). Beneath this coat, the breed’s darkly pigmented skin provides essential protection against harmful ultraviolet (UV) radiation (Silva et al. 2003; Gebremedhin et al. 2023). Together, the combination of a reflective white coat and highly pigmented dark skin offers an effective defense against both heat and UV exposure.

However, this natural protection is compromised in animals affected by skin depigmentation, a condition characterized by the absence of pigment in localized skin areas. Depigmented individuals are more susceptible to sunburn, skin lesions, and heat-related stress issues, which can negatively impact their welfare and productivity. The growing intensity of environmental stressors driven by climate change, such as prolonged exposure to extreme temperatures and high UV radiation, may further aggravate these effects by disrupting melanogenesis, the process responsible for pigment production (Miyamura et al. 2007). As a result, depigmented animals can face early culling, primarily for two reasons: (1) failure to meet breed standards due to altered physical appearance, and (2) increased sensitivity to environmental stress, particularly under pasture-based systems (Vargas et al. 2022).

In addition to disqualifying animals from breed standards and increase the risk of sunburn, skin depigmentation reduces heat tolerance and negatively impacts market value and the well-being of affected animals (Vargas et al. 2022). Recent genetic studies have indicated a potential link between climate-induced stressors and the emergence of novel depigmentation patterns, suggesting that climate change may exacerbate the expression of these phenotypes (Thinda et al. 2021). Additionally, recent reports confirm the appearance of new white patterning in cattle, which may be linked to emerging genetic variants, raising concerns about depigmentation-related issues in these populations (Dai et al. 2024; Petersen et al. 2023). Given that pigmentation serves as a natural defense against UV damage, its loss could negatively impact Nellore’s ability to withstand the harsh conditions of Brazil’s extensive grazing systems (Colombi et al. 2024). Understanding the genetic basis underlying skin depigmentation is therefore essential to developing effective breeding strategies aimed at enhancing Nellore resilience and adaptability under future climatic conditions (Carvalheiro et al. 2019; Colombi et al. 2024).

Disturbances in eyelid pigmentation have been reported in cattle breeds such as Hereford and are considered risk factors for ocular conditions, including squamous cell carcinoma and infectious keratoconjunctivitis (Jara et al. 2022). Similarly, in Fleckvieh cattle, eye-area pigmentation is not only a breed-defining trait but also has important health implications. Specifically, ambilateral circumocular pigmentation (ACOP) is associated with reduced susceptibility to bovine ocular squamous cell carcinoma, the most common malignant tumor affecting cattle (Pausch et al. 2012). Genetic studies suggest that variation in pigmentation is partially explained by genes involved in melanogenesis and skin cancer pathways. Jara et al. (2022) reported moderate heritability for eyelid pigmentation (h^2^ = 0.41) and identified several associated genomic regions. Likewise, Pausch et al. (2012) found that ACOP is highly heritable (h^2^ = 0.79), and identified quantitative trait loci (QTL) and candidate genes associated with pigmentation and eye development, including *KIT*, *MITF*, *PAX3*, and *KITLG*.

In Nellore cattle, depigmentation can be observed in specific parts of the body such as the tail tip and mucosal regions, and as small spots across the body (Salgado 2019). This trait can be evaluated using a binary approach, where individuals are classified as either depigmented (1) or not (0); or using scores defined by specific breeding programs. However, estimates of genetic parameters for skin depigmentation are still scarce in the literature. Salgado (2019) studied an ungenotyped Nellore population from Brazil (N = 75,798) and reported heritability estimates ranging from 0.60 to 0.67 using the threshold model (i.e., liability scale), indicating a strong genetic component and potential for selective breeding against the undesirable phenotype. Moreover, genes such as *LEF1* and *ASIP* have been associated to coat pigmentation and UV protection in cattle (Chen et al. 2023; Flori et al. 2019). Genome-wide association studies (GWAS) can offer an opportunity to identify specific genetic variants and genomic regions associated with depigmentation in Nellore cattle, thereby facilitating a better understanding of the genetic background controlling this trait (Albuquerque et al. 2018; Trigo et al. 2021; Vargas et al. 2022). Therefore, this study aimed to: (1) estimate variance components and genetic parameters for depigmentation in Nellore cattle; (2) identify genetic regions and candidate genes associated with depigmentation; and (3) perform functional analysis in the genomic regions identified to better understand their biological effects.

## Material and methods

Data used in this study were originated from the DeltaGen® breeding program and provided by the Gensys® company (Petrópolis, Porto Alegre, Rio Grande do Sul, Brazil). The DeltaGen® program performs a genetic evaluation for Nellore animals raised in Brazil. Therefore, animal care and use committee approvals were not required, as the data were sourced from preexisting databases.

### Phenotypic data

Phenotypic data were collected by the DeltaGen® program during three evaluation phases: weaning (around 7 months old), yearling (16 months old), and final evaluation (18 months of age), spanning from 1999 to 2023. During these phases, trained professionals collected information on growth, reproductive traits, visual assessment of conformation, precocity, muscularity, and navel condition (CPMU), as well as identified morphological defects. Depigmentation defects were visually assessed using a binary approach (1 for presence and 0 for absence of the defect). Although animals were evaluated at multiple stages (weaning, yearling, and final evaluation), each animal contributed only one observation per defect, as there were no repeated measurements. If an animal was identified with the defect at an early stage, such as at weaning, it was culled and not evaluated again for that trait. As a result, the dataset contains only a single phenotypic record per animal per defect, corresponding to the earliest stage at which the defect was observed.

The raw dataset contained information on 799,672 animals, including both those with and without the defect of interest. Contemporary groups (CG) were created considering birth year and season, sex, farm, management group at weaning and yearling, and date of measurement at weaning and yearling. Contemporary groups with fewer than 10 animals or without phenotypic variability were excluded. Connectedness among CG was verified using the AMC software (Roso and Schenkel 2006), and disconnected groups (n = 16,324) were excluded. After phenotypic quality control, the dataset comprised 182,964 animals raised across 5,629 contemporary groups, with a depigmentation trait incidence of 6.18% (n = 11,310). A descriptive table with the frequency of factors used to create the contemporary groups in the final dataset has been added to the Online Resource 1– Table 1.

### Pedigree and genomic information

The pedigree dataset contained 1,192,464 animals, which spanned up to 12 generations from animals with phenotype. The complete genomic file shared by Gensys® contained genomic information for 68,859 animals. These animals were genotyped using the Neogen®'s 50 K SNP chip (GGP Indicus; Neogen 2021) and imputed to the 777 K density using the Illumina Bovine HD array (Illumina Inc. 2010). Imputation was performed as part of Gensys®'s official evaluation system and the FImpute V3 software (Sargolzaei et al.^2014^), based on a reference population containing 800 Nellore animals with expected accuracy higher than 0.97 (Neves et al. 2014).

Only genotypes for animals with phenotype and/or related to the phenotyped animals were used in this study (n = 28,690). Genotypic quality control (QC) was performed using the PLINK software (Chang et al. 2015) to retain SNPs with genotype call rate > 0.98, minor allele frequency (MAF) > 0.05, and no extreme deviation from the Hardy–Weinberg equilibrium (p < 10⁻^5^). Animals with individual call rate < 0.99 were also removed. Consequently, after quality control, a total of 28,690 genotyped animals (from which 28,655 had both genotype and phenotype), and 385,079 SNPs were available for further analysis.

### Estimation of variance components and genetic parameters

Variance components were estimated using Bayesian inference implemented in the GIBBSF90 + software, which is part of the BLUPF90 family of programs (Misztal et al. 2018). The GIBBSF90 + allows performing Gibbs sampling within a Markov Chain Monte Carlo (MCMC) method to estimate the posterior distributions of the genetic parameters. A total of 500,000 cycles were performed with a burn-in phase of 100,000 cycles and a thin of 50. Convergence assessments were performed using the Geweke et al. (1992), Heidelberger and Welch (1983) criteria, in addition to visual inspection, using the “boa” R package (Smith 2007). In matrix notation, the single-trait threshold model used in this study is defined as:

$$ l = X\beta + Za + e $$

where **l** is the vector of observations in the liability scale, assumed as **l|β,a,**${\upsigma }_{\text{a}}^{2},{\upsigma }_{\text{e}}^{2} \sim \text{ N}$(**Xβ** + **Za**,${\mathbf{I}\upsigma }_{\text{e}}^{2} )$; **β** is the vector of systematic effects of the contemporary groups, assumed as **β** ~ N(0,${\mathbf{I}\upsigma }_{\text{b}}^{2}$), where ${\upsigma }_{\text{b}}^{2}$ has large variances (10^10^) to represent vague prior knowledge; **a** is the vector of direct additive genetic effects, assumed as **a|**${\upsigma }_{\text{a}}^{2},\mathbf{H}$ ~ N(0,$\mathbf{H}{\upsigma }_{\text{a}}^{2}$), where **H** is the relationship matrix that combines pedigree information (**A**; considering up to 5 generations in this study) and genomic (**G**) relationship matrices, and ${\upsigma }_{\text{a}}^{2}$ is the direct additive genetic variance. The $\mathbf{e}$ is the vector of residuals effects, assumed as **e|**${\upsigma }_{\text{e}}^{2}$ ~ N(0,$\mathbf{I}{\upsigma }_{\text{e}}^{2}$), where ${\upsigma }_{\text{e}}^{2}$ is the residual variance and **I** is an identity matrix. The **X** and **Z** are incidence matrices relating the elements to the vectors **β** and **a**, respectively.

An underlying distribution is assumed to the threshold model as follows:

$$ f(y|l_{i} ) = \prod\limits_{i = 1}^{ni} {1(l_{i} < t_{i} )1(y = 0)} + 1(l_{i} > t_{i} )1(y = 1) $$

where y is the binary trait, ${\text{l}}_{\text{i}}$ is the underlying liability of observation *i*; ${\text{t}}_{\text{i}}$ is the threshold that defines the category response for the traits, and n_i_ corresponds to the number of observations.

The inverse of the hybrid pedigree-genomic relationship matrix (**H**^**−1**^), was created as described by Aguilar et al. (2010), i.e.:

$$ H^{ - 1} = A^{ - 1} + \left[ {\begin{array}{*{20}c} 0 \\ 0 \\ \end{array} \;\begin{array}{*{20}c} {} \\ {G^{ - 1} } \\ \end{array} \;\begin{array}{*{20}c} 0 \\ - \\ \end{array} \;\begin{array}{*{20}c} {} \\ {A_{22}^{ - 1} } \\ \end{array} } \right] $$

where ${\mathbf{A}}_{22}^{-1}$ is the inverse of the relationship matrix for the genotyped animals and ${\mathbf{G}}^{-1}$ is the inverse of the genomic relationship matrix described in the first method proposed by VanRaden (2008):

$$ G = \frac{ZZ^{\prime}}{{2\mathop \sum \nolimits_{i = 1}^{m} p_{i} \left( {1 - p_{i} } \right)}} $$

where **Z = **(**M– P**), in which **M** is the SNP incidence matrix, with *m* columns (number of SNP markers) and *n* lines (number of genotyped animals). The elements in **M** were set to 0, 1, and 2 for the genotypes AA, AB, and BB, respectively. The **P** is the matrix with the allele frequencies expressed as 2pi, and p_i_ is the frequency of the *i*th SNP marker. To overcome the software limitation, a random subset of 24,562 genotypes (taken from the 28,655 genotypes available after the quality control) was used to create the **G** matrix.

The heritability in the liability scale was estimated as ${h}_{l}^{2}=\frac{{\sigma }_{a}^{2}}{{\sigma }_{p}^{2}}$, where ${\sigma }_{a}^{2}$ is the genetic additive variance, and ${\sigma }_{p}^{2}$ is the phenotypic variance of the trait. Thereafter, the heritability estimated in the liability scale was converted to the observed scale using the equation originally proposed by Dempster and Lerner (1950) for binary traits, i.e.:

$$ h_{o}^{2} = \frac{{z^{2} h_{l}^{2} }}{{ \propto \left( {1 - \propto } \right)}} $$

where ${\text{h}}_{\text{o}}^{2}$ is the heritability estimate on the observed scale, z is the height (probability density) of the ordinate of the standard normal probability density function at the point corresponding to the threshold between categories, estimated from the incidence ($\propto$) of traits, and ${\text{h}}_{\text{l}}^{2}$ is the heritability estimate on the liability scale.

### Primary genome-wide association study

The ultra-fast generalized linear mixed model-based association analysis for binary traits (fastGWA-GLMM) (Jiang et al. 2021) implemented in GCTA (Yang et al. 2011) was used for the GWAS. This method integrates a generalized linear mixed model (GLMM) into the fastGWA framework, using sparse matrix-based relationship matrix (GRM) algorithms for parameter estimation and association testing (Jiang et al. 2021). Only the animals that contained both phenotypes and genotypes (n = 28,655) were included in the association analyses. The model fitted is defined as:

$$ \log it\left( \mu \right) = x_{s} \beta_{s} + X_{c} \beta_{c} + g $$

where $\mathbf{y}$ is a $\text{n x }1$ vector of binary phenotypes of the depigmentation trait, ${\varvec{\mu}}$ is a vector of ${\mu }_{i}=P\left({y}_{i}=1|{x}_{si},{\text{X}}_{\text{ci}},{g}_{i}\right)$ with ${\mu }_{i}$ representing the probability of an individual $i$ being a case given their genotype ${x}_{si}$, fixed effects (contemporary groups) ${\text{X}}_{\text{ci}}$, and the animals were used as a random genetic effect ${g}_{i}$. The ${{\varvec{x}}}_{{\varvec{s}}}$ is a vector of genotypes of a variant of interest with its effect ${\upbeta }_{\text{s}}$, ${\mathbf{X}}_{\mathbf{c}}$ is the incidence of contemporary groups used as a fixed effect with their corresponding coefficients ${\beta }_{c}$. The $\mathbf{g}$ is a vector of effects that capture genetic and common environment effects shared among related individuals, $\text{g}\sim \text{N}\left(0,\uppi {\upsigma }_{\text{g}}^{2}\right)$ with $\uppi$ being the sparse GRM (i.e., GRM with all the small off-diagonal elements set to zero), and ${\upsigma }_{\text{g}}^{2}$ being the corresponding variance component.

The fastGWA-GLMM method consists of two main steps. First, parameter estimation using a computationally efficient grid search-based algorithm (Jiang et al. 2021). Second, association testing is performed via a score test for each variant:

$$ T_{score} = x_{s}^{T} (y - \mu )\;with\;{\text{var}} (T_{score} ) = x_{s}^{T} Px_{s} $$

$$ \frac{{T_{score}^{2} }}{{{\text{var}} (T_{score} )}} \sim X_{d.f. = 1}^{2} $$

where $\mathbf{P}={\mathbf{V}}^{-1}-{\mathbf{V}}^{-1}{\mathbf{X}}_{\text{c}}{\left({\mathbf{X}}_{\text{c}}^{\text{T}}{\mathbf{V}}^{-1}{\mathbf{X}}_{\text{c}}\right)}^{-1}{\mathbf{X}}_{\text{c}}^{\text{T}}{\mathbf{V}}^{-1}$ with $\mathbf{V}={\mathbf{W}}^{-1}+\uppi {\upsigma }_{\text{g}}^{2}$ and **W** is a diagonal matrix, i.e., ${\text{W}}_{\text{ii}} = {\upmu }_{\text{i}}\left(1-{\upmu }_{\text{i}}\right)$. The $\mathbf{P}$ is an $\text{nxn}$ projection matrix, which is dense despite $\pi$ being sparse. The Bonferroni correction method (α = 0.05) was used to adjust for multiple-testing correction based on the number of independent chromosomal segments, the average length of a chromosome, and the number of chromosomes at the chromosome-wide level (Corbin et al. 2012). This methodology accounts for multiple testing and adjusts the significance thresholds of the SNPs accordingly (Goddard et al. 2011).

The proportion of genetic variance explained by each chromosome was calculated following the approach described by Wang et al. (2012):

$$ \frac{{Var_{{(a_{i} )}} }}{{\sigma_{a}^{2} }} \times 100\% = \frac{{Var\left( {\sum\limits_{j = 1} {z_{j} \overset{\lower0.5em\hbox{$\smash{\scriptscriptstyle\frown}$}}{u}_{j} } } \right)}}{{\sigma_{a}^{2} }} \times 100\% $$

where ${\text{a}}_{\text{i}}$ is the genetic value for the SNPs on i^th^ chromosome; ${\upsigma }_{\text{a}}^{2}$ is the total additive genetic variance; z_j_ is genotype vector (0, 1, or 2) for j^th^ SNP; and ${\widehat{\text{u}}}_{\text{j}}$ is the estimated effect for the j^th^ SNP. The total variance explained by each chromosome was obtained by summing the contributions of all SNPs located on that chromosome. These values were then normalized to express each chromosome’s contribution as a percentage of the total variance explained by all SNPs.

#### Conditional genome-wide association analysis

To investigate the independence of association signals and evaluate potential confounding effects from the strong signal observed on BTA22, we performed a conditional GWAS using the fastGWA-GLMM framework implemented in GCTA. The conditional analysis was motivated by several factors: (1) the presence of a highly significant association signal on BTA22 in the *MITF* region, a well-established candidate gene for pigmentation traits across multiple species (Negro et al. 2017; Petersen et al. 2023; Wen et al. 2024); (2) the need to determine whether associations on other chromosomes were independent or potentially driven by linkage disequilibrium with the BTA22 signal; and (3) the importance of characterizing the true genetic architecture underlying depigmentation in Nellore cattle. Therefore, the SNP located at position 31,681,219 on BTA22 (based on the ARS-UCD1.3 bovine genome assembly) was selected as the conditioning covariate based on the following criteria: (1) it exhibited the strongest association signal on BTA22 in the primary GWAS; (2) it is located within the *MITF* gene region, specifically in the promoter region known to regulate melanocyte development and function; and (3) it explained the largest proportion of the genetic variance for depigmentation. The conditional model is defined as:

$$ \log it(\mu ) = x_{s} \beta_{s} + X_{c} \beta_{c} + g + \varphi MITF $$

where all parameters are as defined in the primary GWAS model, with the addition of *MITF* representing the genotype dosage of the conditioning SNP (coded as 0, 1, or 2 for the number of copies of the reference allele) and φ representing its additive genetic effect (regression coefficient). This approach treats the conditioning SNP as a linear covariate, effectively removing the genetic effect of the conditioning SNP and allowing for the detection of independent association signals that may have been masked or confounded in the primary analysis. All other analytical parameters remained identical to the primary GWAS.

### Gene Annotation, QTL Identification, and Functional Analyses

The significant markers associated with the depigmentation trait from both the primary and conditional GWAS were subjected to gene annotation and functional enrichment analyses. Gene annotation was performed using the GALLO package (Fonseca et al. 2020) implemented in R (R Core Team 2024). For this purpose, a genomic window of 100 kb upstream and downstream from each significant SNP was considered, based on the ARS-UCD1.3 bovine genome assembly (Rosen et al. 2020). Identified positional candidate genes were then analyzed using the gprofiler2 package (Kolberg et al. 2020) to perform functional enrichment. Enrichment was assessed for Gene Ontology (GO) categories, including Biological Processes (BP), Molecular Functions (MF), and Cellular Components (CC), as well as for Kyoto Encyclopedia of Genes and Genomes (KEGG) pathways. Additionally, to explore potential biological interactions and regulatory networks, gene network analysis was performed using NetworkAnalyst 3.0 (Zhou et al. 2019).

In parallel, QTL annotation was performed using the same set of significant SNPs and 100 kb window strategy, using the GALLO package (Fonseca et al. 2020) and the ARS-UCD1.3 genome assembly as reference (Rosen et al. 2020). The annotated QTL regions were then cross-referenced with trait-associated QTLs previously reported in cattle to identify overlaps and potential biological relevance. For this purpose, QTL information was retrieved from the Animal QTL Database (Hu et al. 2013). Enrichment analysis of the annotated QTLs was performed to assess their over-representation within specific trait categories, offering further insight into the potential broader phenotypic effects associated with depigmentation-related genomic regions.

## Results and discussion

This study focuses on skin depigmentation in Nellore cattle, which is characterized by the loss of melanin in specific skin regions, resulting in lighter patches. Importantly, this condition differs from the general white color. According to Grandin and Deesing (2022), depigmentation involves the reduction or absence of melanin in localized areas, but the animal still retains functional melanocytes that produce melanin in other parts of the skin or coat. In contrast, white color refers to the complete absence of melanin in the skin or coat, leading to a uniform white appearance. This condition is typically caused by genetic mutations that prevent melanocytes from producing melanin, despite the cells being present (Fontanesi et al. 2010). For instance, in breeds such as Holstein and Brown Swiss, mutations in the *KIT* and *MITF* genes lead to a complete lack of pigmentation, resulting in animals that are fully white (Milia et al., 2024). In Nellore cattle, however, depigmentation affects the skin and mucous membranes instead coat color.

Understanding the genetic architecture of depigmentation and identifying the candidate genes and QTL associated with this undesirable phenotype can help breeders minimize culling and economic losses. From an animal perspective, preserving skin pigmentation is essential, as they contribute to the adaptability of Nellore cattle in tropical environments. Additionally, estimating genetic parameters for this trait can help to develop optimal selection strategies, and determining the best approach for incorporating this trait into breeding programs.

## Descriptive statistics

**Fig. 1:**
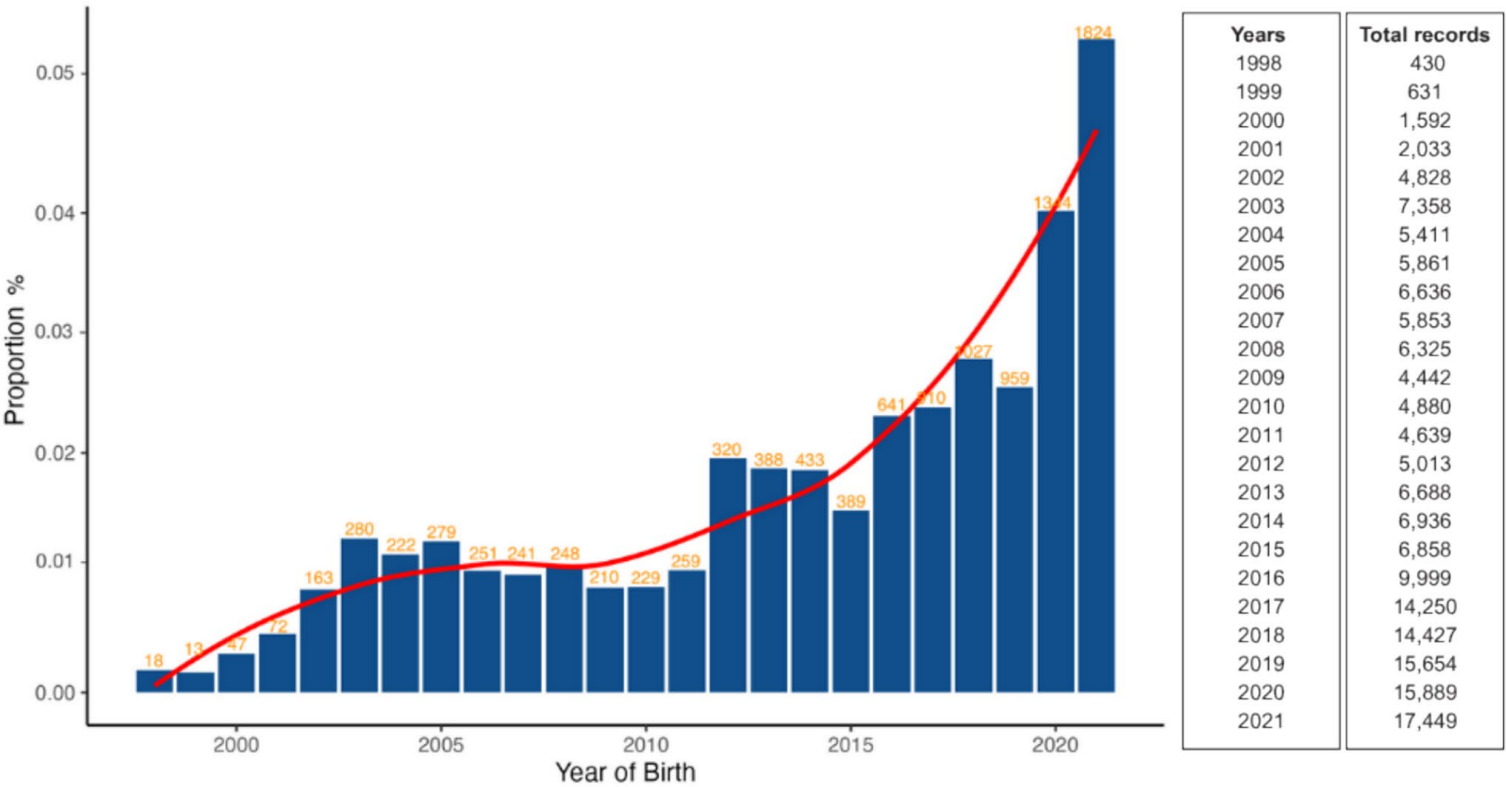
Incidence of depigmentation in the Nellore cattle population over the years. The height of each bar represents the proportion of animals affected by depigmentation in a given year, with the number of depigmented animals indicated at the top of each bar. The total number of animals evaluated each year is shown on the right side of the figure. Notably, 2021 is the last year in the dataset for which complete information was available across the entire productive cycle

Figure 1 shows the incidence of depigmentation in the Nellore cattle population over time. A clear upward trend is observed in the occurrence of depigmentation, with a substantial increase in both the number of affected animals and the number of farms reporting cases. For instance, in 1998, only two farms recorded depigmentation, whereas by 2021, this number had risen to 53. This increase likely reflects not only improved data collection and broader herd coverage over the years but also growing awareness of the phenotype among producers and technicians. Nevertheless, the consistent rise across time suggests that external factors may be influencing the prevalence of this condition.

One plausible explanation for the observed increase in incidence of depigmentation is the rising exposure to solar UV radiation. Although our data do not directly assess UV levels, the temporal pattern is consistent with previously documented increases in surface UV intensity (e.g., Tang et al. 2024). Melanin offers photoprotective benefits, and individuals with darker pigmentation are generally more resistant to UV-induced damage (Gloger’s rule; Roulin 2014). In this context, genes associated with skin pigmentation may not only mitigate susceptibility to depigmentation but also enhance overall resilience to environmental stressors. These findings support the idea that selection for pigmentation traits may offer adaptive advantages in the face of changing environmental conditions.

## Variance components and genetic parameters

Variance components and genetic parameters, along with their 95% highest posterior density (HPD) intervals, estimated for depigmentation are shown in Table 1. The heritability transformed to the observed scale is 0.12.

**Table 1:** Genetic parameters estimated for depigmentation in Nellore cattle

Genetic parameters	Liability scale	HPD interval
${\sigma }_{a}^{2}$	1.20	1.05–1.35
${\sigma }_{e}^{2}$	1.00	1.00–1.02
${h}^{2}$	0.54	0.51–0.57

${\sigma }_{a}^{2}$: Additive genetic variance; ${\sigma }_{e}^{2}$: residual variance; ${h}^{2}$: heritability; HPD: their 95% highest posterior density intervals

The difference in scale observed in genetic parameters can lead to misinterpretations, particularly when comparing estimates across studies or making selection decisions (Hidalgo et al. 2024a, b). This occurs because, when working with binary traits, their expression on the observed scale (0,1) introduces measurement error, which inflates environmental variance and causes heritability estimates to depend on trait incidence (Falconer and Mackay 1996). To address this, binary traits are converted to the liability scale, where heritability estimates are independent of incidence and better reflect the underlying genetic variation (Dempster and Lerner 1950; Falconer and Mackay 1996; Hidalgo, et al. 2024a, b). However, heritability on the observed scale remains relevant for practical breeding applications, as selection response ultimately occurs in the observed population (Gianola 1982; Robertson and Lerner 1949). On the liability scale, we observe a heritability of 0.54 (Table 2), which is considered high (> 0.4). However, when converted to the observed scale, the heritability is 0.12, which is low (< 0.2). These results are lower than those reported by Salgado (2019), who studied an ungenotyped Nellore population from Brazil (N = 75,798), and reported heritability estimates ranging from 0.60 to 0.67 using the threshold model (i.e., on the liability scale).

**Table 2:** Candidate genes associated with depigmentation in Nellore cattle

Gene	Chromosome	Genomic Region (Start–End; bp)	SNP associated
*KIT*	6	70,166,692–70,254,044	34
*EDNRB*	12	53,038,377–53,068,132	14
*MITF*	22	31,616,846–31,857,969	21
*GNAI2*	22	50,099,986–50,120,522	1

SNP associated: number of SNP markers significant associated with the candidate gene; bp: base par

Studies specifically addressing depigmentation in cattle are scarce. Jara et al. (2022) analyzed eyelid pigmentation in 4,929 Hereford cattle using categorical scores (0%, 25%, 50%, 75% and 100% pigmented) and reported heritability estimate of 0.41. In horses, Vitiligo-like depigmentation traits has been extensive studied, often involving genes such as *KIT* and *MITF*, with heritablility estimates ranging from 0.09 to 0.63 using different scores of pigmentation (Curik et al. 2013; Druml et al. 2021). In pigs, a GWAS of iris pigmentation performed using 897 animals based on a scoring-based phenotype estimated SNP heritability at 0.11 for depigmented eyes (Moscatelli et al. 2020). Our lower value in heritability does not mean that the trait will not respond to selection, but rather that the genetic progress will be slower than the genetic progress observed for highly heritable traits. As noted by Falconer and Mackay (1996) and Harris and Johnson (1999), traits with lower heritability will still respond to selection, but the rate of genetic improvement will be less than that seen in traits with higher heritability. Similarly, Hill (2014) discusses how the response to selection is inversely related to the heritability of a trait, with lower heritability leading to slower genetic progress.

## Primary GWAS results

**Fig. 2:**
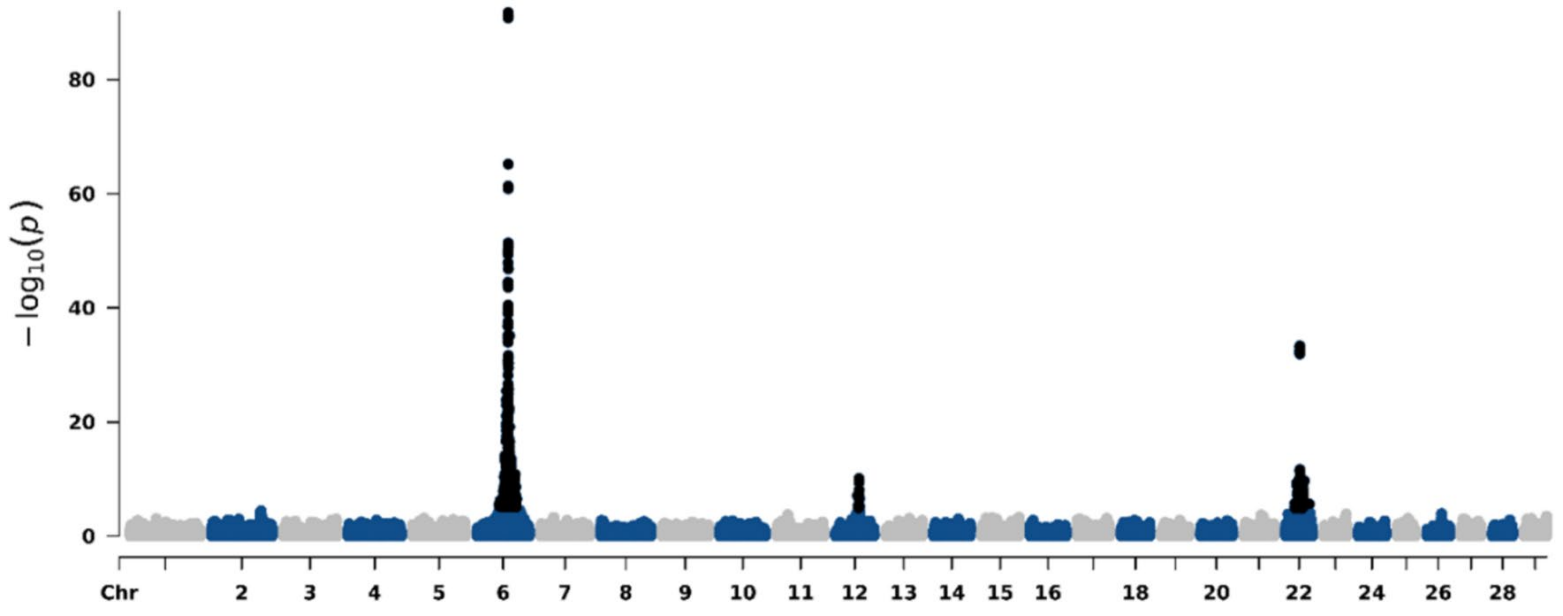
Manhattan plot for the depigmentation defects in the Nellore cattle population. Black dots represent the significant markers

Figure 2 shows the Manhattan plot for the depigmentation in Nellore cattle. A total of 1,044 significant SNP markers (p < 0.05 after Bonferroni correction) associated with depigmentation defects in Nellore indicine beef cattle were identified in this study. These markers were distributed across three chromosomes (i.e., BTA6, BTA12 and BTA22).

The significant SNPs found on BTA6, BTA12 and BTA22 explained 24%, 4% and 6% of the genetic variance of depigmentation, respectively. The top marker that appears in the BTA6 is located at the position 70,327,151 bp (based on the ARS-UCD1.3 bovine genome assembly; Rosen et al. 2020). In total, 316 genes were identified in these regions, of which 205 were protein-coding, 90 long non-coding RNAs, 2 microRNAs, 3 pseudogenes, 15 small nuclear RNA and 1 small nucleolar RNAs. Among the identified candidate genes, *KIT, EDNRB, MITF,* and *GNAI2* were located near significant SNPs and appear to be directly involved in the pigmentation processes (Table 2). The table including all positional candidate genes identified for depigmentation can be found in the Online Resource 1 Table 2.

The significant associations on BTA6, BTA12, and BTA22 align with previous studies on white spotting in Holstein–Friesian dairy cattle. For instance, Jivanji et al. (2019) analyzed 885 outbred dairy bulls, 1,389 outbred dairy cows, and 699 cross cows from an experimental pedigree. The authors used video footage to score animals for the presence or absence of white on their coat and quantified the proportion of white spotting. They performed a GWAS study and a structural variant analysis, characterizing white spotting as a quantitative trait controlled by multiple QTL. Their study identified the top 10 variants for each significant QTL detected in the GWAS analysis, with the strongest associations found on chromosomes BTA22, BTA6 and BTA2. Notably, two genomic regions located in these chromosomes (i.e., regions near the rs451683615 and rs209784468 SNPs on BTA6 and BTA22, respectively), overlapped with our findings—strengthening the evidence that these genomic regions may be involved in skin depigmentation in Nellore cattle.

## Conditional GWAS results

**Fig. 3:**
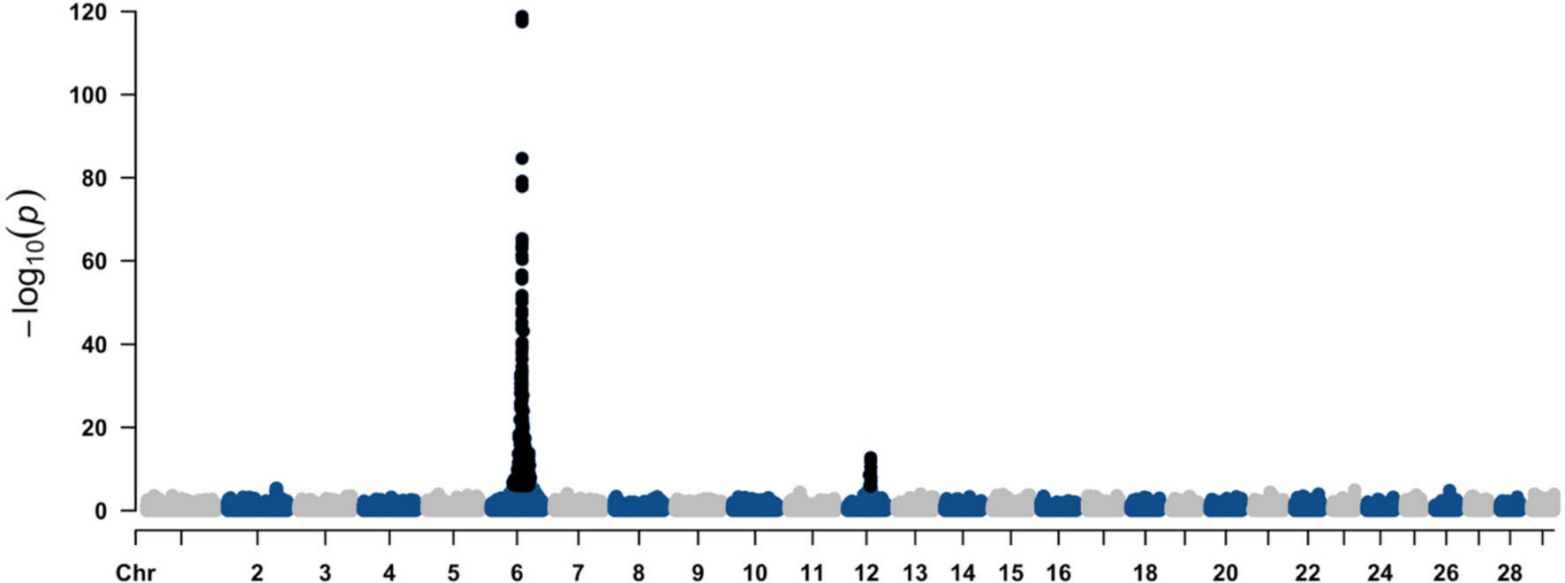
Manhattan plot for the depigmentation defects in the Nellore cattle population after conditional GWAS. Black dots represent the significant markers

To evaluate the independence of association signals and investigate potential confounding effects from the strong *MITF* signal on BTA22, we performed a conditional GWAS analysis. Figure 3 shows the Manhattan plot for the depigmentation defects in the Nellore cattle population after the conditional GWAS. In summary, the conditional GWAS identified 1,011 significant SNPs (p < 0.05 after Bonferroni correction), representing a reduction of 33 SNPs compared to the primary analysis. Most notably, the conditioning procedure completely eliminated the association signal on BTA22, with no SNPs in the *MITF* region remaining significant after accounting for the conditioning SNP. This finding suggests that the original BTA22 signal was largely driven by a single major locus rather than multiple independent variants in the region.

The significant SNPs found on BTA6 and BTA12 after the conditional GWAS explained 26.5% and 4% of the genetic variance of depigmentation, respectively. These markers mapped to 234 genes, including 129 protein-coding genes, 84 long non-coding RNAs (lncRNAs), 7 microRNAs, 1 pseudogene, 12 small nuclear RNAs (snRNAs), and 1 small nucleolar RNA (snoRNA). Notably, candidate genes such as *KIT* and *EDNRB* remained, while those previously identified on chromosome 22 were no longer detected.

## Gene network interactions, QTL identification, and functional analyses

**Fig. 4:**
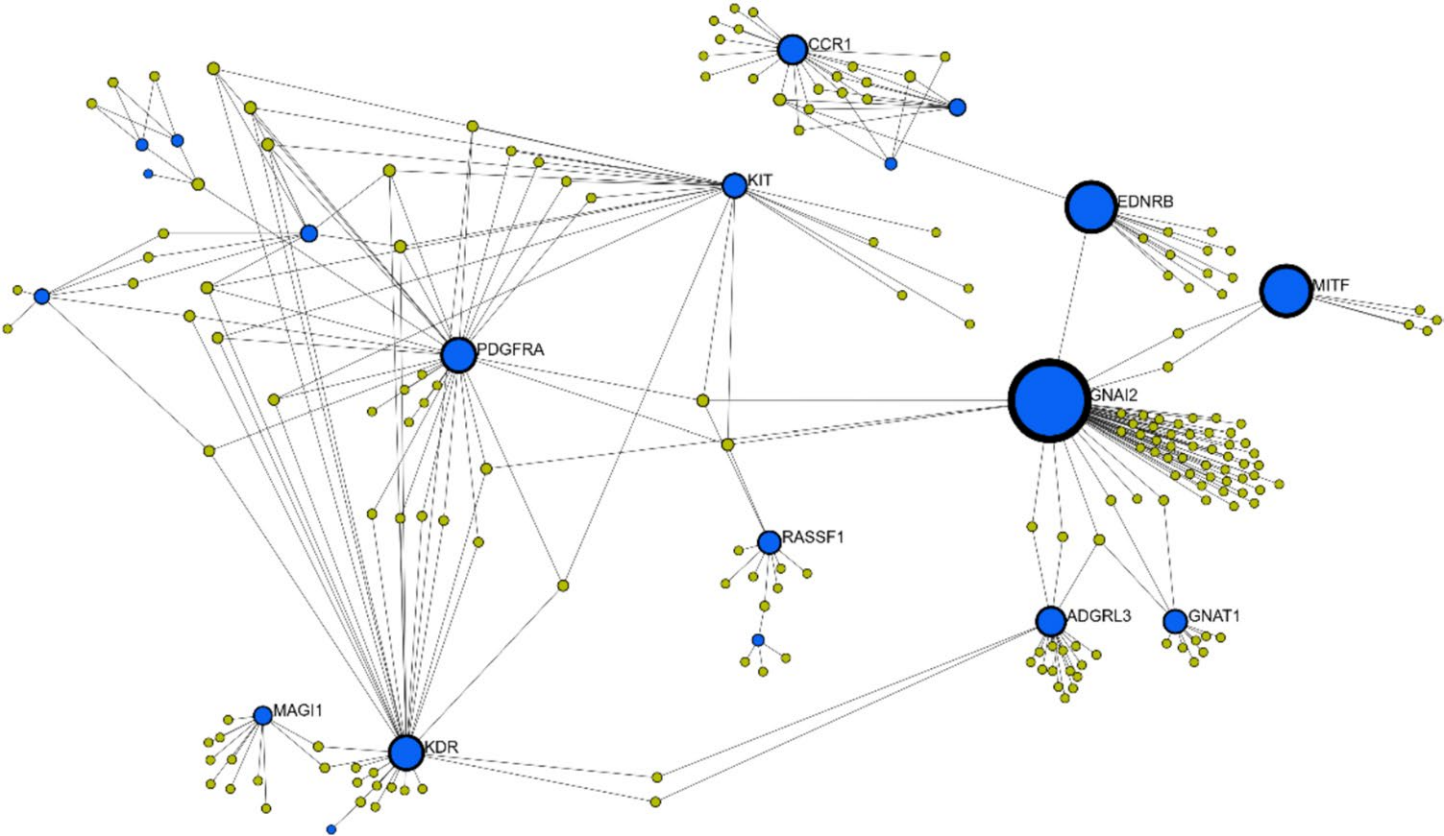
Gene network interactions, where the blue dots represent the gene, and the yellow dots represent the proteins. Image generated using the Network Analyst (Zhou et al. 2019), and results from the primary GWAS

Gene network, QTL identification, and functional analyses were performed using results from both primary and conditional GWAS. The interactions among all the identified genes indicate that complex mechanisms and pathways could contribute to variation in skin pigmentation in Nellore cattle (Fig. 4). For instance, *MITF* is located in the BTA22 and was associated with 21 significant SNPs. Dai et al. (2024) also identified *MITF* as a key gene in buffaloes, based on the whole-genome sequencing data of solid and spotted animals, and highlighted the involvement of *KIT* in the development of this phenotype. The *MITF* gene*,* or microphthalmia-associated transcription factor, is essential for melanocyte survival, proliferation, differentiation, and pigmentation by regulating *C-KIT, Tyr, Dct*, and anti-apoptotic genes (Wen et al. 2024). Specifically, *C-KIT* (Kit Proto-Oncogene, Receptor Tyrosine Kinase) encodes a receptor for melanocyte migration from the dermis to the epidermis (Cui and Man 2023). Tyrosinase (*Tyr*) catalyzes the first step of melanin biosynthesis, converting tyrosine to dopaquinone, essential for eumelanin and pheomelanin production (Sarini et al. 2022; Cui and Man 2023). The *Dct* gene further contributes by converting dopachrome to DHICA, aiding pigment formation and protecting melanocytes from oxidative stress (Cui and Man 2023). Together, the coordinated regulation of these genes by *MITF* underpins proper melanocyte function and normal pigmentation. Disruption in any component of this network can lead to depigmentation phenotypes (Wen et al. 2024).

Previous studies have reported white spotting phenotypes linked to the *KIT* and *MITF* genes in dairy and beef cattle (Jakaria et al. 2023; Jivanji et al. 2019; Maciel et al. 2024; Petersen, et al. 2023; Sarini et al. 2023), horses (Negro et al. 2017; Rosa et al. 2022), llamas and alpacas (Anello et al. 2022), ducks (Zhang et al. 2023), and camels (Holl et al. 2017). The overlap of genomic regions across species suggests a conserved genetic basis for pigmentation variation. In humans, mutation in the *KIT* gene is associated with piebaldism, a rare genetic disorder characterized by depigmented patches throughout the body (Budair 2024). Variants in *EDNRB* disrupted melanocyte development and function, leading to hypopigmentation in affected individuals (Wang et al. 2023). In horses, two adjacent SNPs in heterozygosis promotes overo white markings and in homozygosis a complete white foal that dies after birth due to deficiency in intestine innervation (Metallinos et al. 1998). Interestingly, the *GNAI2* gene, or G Protein Subunit Alpha I2, has only been identified in the primary GWAS, i.e., before accounting for the *MITF* region in the statistical model. This suggests that *GNAI2* may have been identified due to the linkage disequilibrium in the region, not necessary due to its independent effect on depigmentation. Regardless, *GNAI2* has been shown to promote cell proliferation while inhibiting the apoptosis process (Hu et al. 2021), and its upregulation enhances melanocyte proliferation (Wang et al. 2024). Given that *GNAI2* is involved in the regulation of melanocyte-stimulating hormone (MSH) signaling, which is essential for melanin production, disruption in *GNAI2* function may alter MSH responses, reducing melanin synthesis and contributing to skin depigmentation (Wang et al. 2024). Further studies to better understand the role of the *GNAI2* gene in depigmentation are needed.

The pathways identified in this study that seem to be related to pigmentation, based on the primary GWAS results, are melanogenesis (KEGG:04916); cellular response to UV-B (GO: 0071493); vascular endothelial growth factor binding (GO: 0038085) and receptor activity (GO: 005021); epidermal growth factor receptor binding (GO: 0005154); ascorbate and aldarate metabolism (KEGG: 00053); and retinol metabolism (KEGG: 00830) (Table 3). The complete list of Gene Ontology terms identified using the primary GWAS results is shown in the Online Resource 1– Table 3.4 Notably, gene ontology performed using the results from the conditional GWAS did not identify any terms explicitly related to pigmentation or pigment biosynthesis. The disappearance of pigmentation-related GO terms after conditioning aligns with the elimination of the *MITF* signal, as this transcription factor is central to most canonical pigmentation pathways. The pathways that remained significant after conditioning included various metabolic processes, cell signaling pathways, and developmental processes that may represent upstream regulators of pigmentation or alternative mechanisms leading to melanocyte dysfunction. The full list of annotated genes and enriched ontology terms using the conditional GWAS results is provided in the Online Resource 1 (Tables 4 and 5).

**Table 3:** Gene Ontology terms for the candidate genes annotated for depigmentation trait using the primary GWAS results

Functional terms	Source	Description of function	Genes
GO:0071493	GO:BP	cellular response to UV-B	ENSBTAG00000000484,ENSBTAG00000000483,ENSBTAG00000052388
GO:0038085	GO:MF	vascular endothelial growth factor binding	ENSBTAG00000000782,ENSBTAG00000007173
GO:0005021	GO:MF	vascular endothelial growth factor receptor activity	ENSBTAG00000000782,ENSBTAG00000007173
GO:0005154	GO:MF	epidermal growth factor receptor binding	ENSBTAG00000004052,ENSBTAG00000010273,ENSBTAG00000018134
KEGG:00053	KEGG	Ascorbate and aldarate metabolism	ENSBTAG00000059934,ENSBTAG00000053565,ENSBTAG00000053282,ENSBTAG00000064079,ENSBTAG00000039991,ENSBTAG00000058539
KEGG:00830	KEGG	Retinol metabolism	ENSBTAG00000059934,ENSBTAG00000053565,ENSBTAG00000053282,ENSBTAG00000064079,ENSBTAG00000039991,ENSBTAG00000058539
KEGG:04916	KEGG	Melanogenesis	ENSBTAG00000002699,ENSBTAG00000005299,ENSBTAG00000006679,ENSBTAG00000020645

*GO* Gene ontology, *BP* Biological process, *CC* Cellular component, *MF* Molecular function, *KEGG* Kyoto encyclopedia of genes and genomes

In our study, Gene Ontology (GO) terms pointed to key biological processes related to pigmentation. The melanogenesis pathway (KEGG:04916) suggests a strong involvement of genes that regulate melanocyte function and pigment production (Suryaningsih 2020). Lin et al. (2017) demonstrates that the cellular response to UV-B (GO:0071493) is important because UV-B exposure can influence melanin production. An impaired response to UV-B could contribute to reduced melanin synthesis and increased susceptibility to depigmentation. Vascular endothelial growth factor binding (GO:0038085) and receptor activity (GO:005021) are also implicated, as VEGF plays a role in skin and hair follicle angiogenesis, indirectly influencing melanocyte function and pigmentation (Alsohaimi 2019). Additionally, epidermal growth factor receptor binding (GO:0005154) indicates that the epidermal growth factor receptor (EGFR) is involved in skin homeostasis and pigmentation (Alsohaimi 2019); mutations or altered EGFR activity have been linked to pigmentation disorders. Ascorbate and aldarate metabolism (KEGG:00053) suggest that Vitamin C (ascorbate) inhibits melanin synthesis by reducing tyrosinase activity, an enzyme that potentially led to skin lightening or depigmentation (Sanadi and Deshmukh 2020). Finally, retinol metabolism (KEGG:00830) shows that retinoids (Vitamin A derivatives) regulate skin cell turnover and melanogenesis, and excessive retinoid activity can inhibit melanin production, contributing to depigmentation (Ortonne 2006).

**Fig. 5:**
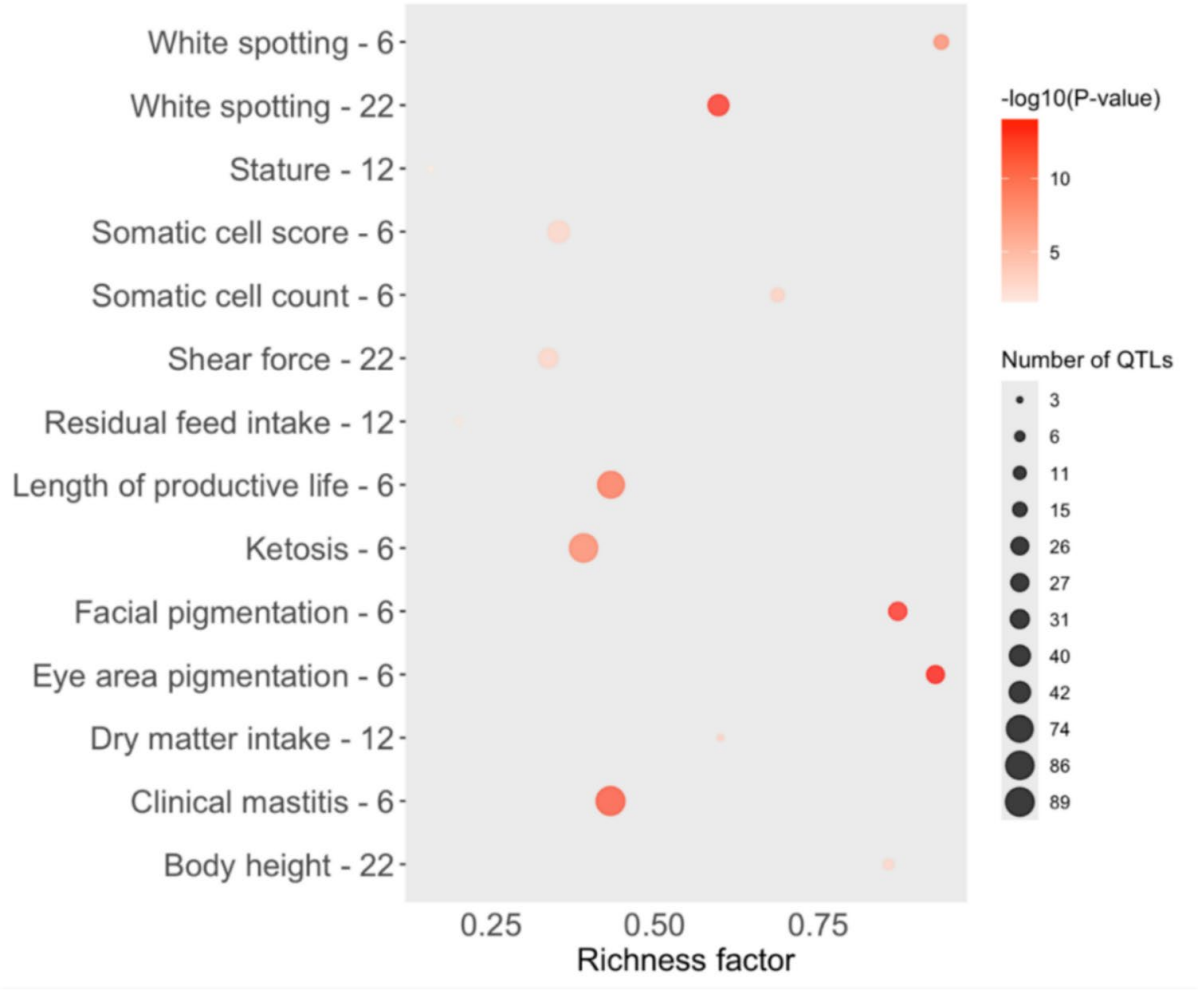
Quantitative trait loci (QTL) enrichment for the significant regions for the depigmentation trait using the primary GWAS results

The QTL enrichment analysis performed using the primary GWAS results indicates significant findings for white spotting in BTA6 and BTA22, and for eye area pigmentation and facial pigmentation in BTA6. Other QTL types identified are shown in Fig. 4. Traits such as eye area pigmentation not only exhibit strong statistical significance (as shown by darker red color) but also high richness and a large number of associated QTLs, suggesting a polygenic basis with potential involvement of dense regulatory regions (Jahuey-Martínez et al. 2024). The co-enrichment of pigmentation traits on BTA6 points to shared genetic mechanisms, possibly involving pleiotropic loci or common regulatory pathways. These findings pinpoint genomic regions potentially involved in pigmentation patterns, suggesting the presence of regulatory elements or genes influencing depigmentation traits in Nellore cattle. QTL enrichment analysis performed using the results from the conditional GWAS still indicate significant associations for white spotting, eye area pigmentation, and facial pigmentation on BTA6. The persistence of these QTL associations after conditioning supports the independence of the BTA6 signal and its distinct biological role in pigmentation control (Fig. 5). Details about the QTL enrichment analysis results from the conditional GWAS are included in the Online Resource 1– Fig. 1.

## Implications for genetic architecture

The conditional GWAS results provide important insights into the genetic architecture of depigmentation in Nellore cattle. The findings suggest a model where depigmentation is controlled by at least two major independent pathways: (1) a primary pathway involving *MITF*-mediated transcriptional regulation of melanogenesis, represented by the major locus on BTA22; and (2) a secondary pathway involving *KIT*-mediated melanocyte development and function, represented by the independent signal on BTA6. This multi-pathway model has important implications for breeding strategies. For instance, while selection against the major *MITF* variant would likely reduce the overall incidence of depigmentation, the presence of an independent loci means that complete elimination of the phenotype would require consideration of multiple genomic regions. Furthermore, the identification of independent pathways suggests that different forms or severities of depigmentation may be associated with different genetic mechanisms. Animals carrying variants in both pathways might exhibit more severe phenotypes than those affected by single pathways, although this hypothesis would require further investigation with more detailed phenotypic characterization.

The identification of multiple independent pathways for depigmentation also has implications for understanding gene-environment interactions and the adaptive significance of pigmentation variation. The temporal increase in depigmentation incidence observed in our study, potentially linked to increasing UV radiation intensity, suggests that environmental factors may differentially affect the expression of variants in different pathways. The *MITF* pathway, being primarily involved in transcriptional regulation of melanogenesis, might be more sensitive to environmental factors that directly affect gene expression, such as UV radiation, oxidative stress, or nutritional factors. In contrast, the *KIT* pathway, being more involved in developmental processes, might be less environmentally labile but could show stronger effects during critical developmental windows. This differential environmental sensitivity could have important implications for breeding strategies in the context of climate change. If UV radiation intensity continues to increase as predicted, variants in pathways that confer greater environmental resilience might become increasingly valuable. Understanding which pathways provide better protection against environmental stressors could inform breeding decisions aimed at maintaining animal welfare and productivity under changing climatic conditions.

## Limitations and future directions

While this study provides important insights into the prevalence and potential genetic basis of depigmentation in Nellore cattle, several limitations should be acknowledged, and future research directions proposed. One key limitation lies in the nature of the depigmentation phenotype itself, which is inherently difficult to record with precision. Depigmentation may develop progressively and may not be present or detectable at the time of routine phenotypic evaluations. In our dataset, animals were primarily assessed at specific stages of the production cycle, which may not capture late-onset cases. As a result, animals that had not yet expressed the phenotype might have been misclassified as unaffected. This misclassification likely leads to underestimation of both the prevalence and heritability of the trait, as well as potential attenuation of genetic associations due to phenotypic noise.

Moreover, the current recording system does not capture detailed information on the anatomical location or extent of depigmentation. Preliminary observations suggest that depigmentation can affect various regions of the body, such as the muzzle, periocular area, ears, or ventral skin, and that the pattern and severity may vary between individuals. It is plausible that these variations represent distinct but genetically correlated traits, each influenced by different biological mechanisms. Treating depigmentation as a single binary trait may therefore oversimplify the phenotype and obscure meaningful biological heterogeneity. Future studies should aim to develop standardized protocols for detailed phenotyping, including age of onset, anatomical distribution, and severity grading, which would facilitate deeper investigations into the developmental and genetic underpinnings of the condition.

Finally, while this study focused on depigmentation in the context of environmental stressors such as UV radiation, future work should explore potential gene–environment interactions in more depth. Longitudinal monitoring of animals exposed to varying environmental conditions (e.g., different UV indices, altitudes, or management systems) could help clarify how external factors influence phenotypic expression and interact with genetic predispositions. Experimental studies or controlled exposure trials could also be valuable for testing causality and understanding the biological mechanisms involved. Such efforts will be crucial for improving the accuracy of genetic evaluations and informing breeding strategies aimed at maintaining both performance and adaptability in changing environments.

## Conclusion

The heritability estimated for depigmentation in Nellore cattle was 0.54 on the liability scale and 0.12 on the observed scale, indicating an opportunity to decrease the proportion of affected animals in the population using genetic selection. Our findings support the involvement of *MITF, KIT* and *EDNRB* in the depigmentation phenotype, consistent with previous studies in other breeds and species. The gene ontology analysis highlighted biological processes related to melanogenesis, pigmentation, and hypopigmentation phenotypes, while the QTL enrichment analysis identified significant associations on BTA6 and BTA22. These findings enhance our understanding of its genetic architecture, suggest the potential for selection strategies and development causative mutation markers to reduce its incidence while ensuring animal welfare and economic viability.

## Supplementary Information

Below is the link to the electronic supplementary material.

Supplementary file1 (DOCX 234 KB)

## Data Availability

No datasets were generated or analysed during the current study.
