# Supplementary material for: Beyond black and white: dissecting the genetic basis of skin depigmentation in Nellore cattle
Source: Mamm Genome. 2025 Aug 4;36(4):1126–40. doi: 10.1007/s00335-025-10153-9 (PMC12578768; doi:10.1007/s00335-025-10153-9)
Supplement: Supplementary file 1 — Supplementary file1 (DOCX 234 KB) [file 335_2025_10153_MOESM1_ESM.docx]

**Beyond Black and White: Dissecting the Genetic Basis of Skin Depigmentation in Nellore Cattle**

*Mammalian Genome*

Milena A. F. Campos: Escola de Medicina Veterinária e Zootecnia, Universidade Federal da Bahia, Salvador, Bahia, Brazil and Department of Animal Sciences, Purdue University, West Lafayette, Indiana, USA, [mapareci@purdue.edu](mailto:mapareci@purdue.edu).

Hinayah Rojas de Oliveira: Department of Animal Sciences, Purdue University, West Lafayette, Indiana, USA, [hinayah@purdue.edu](mailto:hinayah@purdue.edu).

Gregorio M. F. de Camargo: Escola de Medicina Veterinária e Zootecnia, Universidade Federal da Bahia, Salvador, Bahia, Brazil [gregorio.camargo@ufba.br](mailto:gregorio.camargo@ufba.br).

Henrique A. Mulim: Department of Animal Sciences, Purdue University, West Lafayette, Indiana, USA, [hmulim@purdue.edu](mailto:hmulim@purdue.edu).

Diercles Francisco Cardoso: Gensys® Company, Petrópolis, Porto Alegre, Rio Grande do Sul, Brazil [gensys.diercles@gmail.com](mailto:gensys.diercles@gmail.com).

Raphael Bermal Costa: Escola de Medicina Veterinária e Zootecnia, Universidade Federal da Bahia, Salvador, Bahia, Brazil [raphael.bermal@ufba.br](mailto:raphael.bermal@ufba.br).

*Corresponding author: [hinayah@purdue.edu](mailto:hinayah@purdue.edu)

**Table 1.** Descriptive analysis of the animals recorded in each phase and the factors considered as fixed effect in the contemporary groups.

| Category |  | Frequency |
| --- | --- | --- |
| Total number of animals recorded at weaning |  | 183,003 |
| Total number of animals recorded at yearling |  | 152,527 |
| Total number of animals recorded at final evaluation |  | 37,228 |
| Total number of depigmented animals at weaning |  | 9,310 |
| Total number of depigmented animals at yearling |  | 1,435 |
| Total number of depigmented animals at final evaluation |  | 565 |
| Birth year | 1998-2024 | 26 |
| Season of Birth |  | 4 |
| Sex |  | 2 |
|  | Male | 102,940 |
|  | Female | 80,063 |
| Number of farms at weaning |  | 87 |
| Number of farms at yearling |  | 87 |
| Number of management groups at weaning |  | 781 |
| Number of management groups at yearling |  | 386 |
| Number of days of measurement at weaning |  | 1,596 |
| Number of days of measurement at yearling |  | 1,156 |

**Table 2.** Positional candidate genes located near the regions identified in this study

| Gene | Chromosome | Genomic Region (Start – End) | | gene_id | gene_biotype |
| --- | --- | --- | --- | --- | --- |
| *YIPF7* | 6 | 63238860 | 63290332 | ENSBTAG00000001603 | protein_coding |
| *GUF1* | 6 | 63290166 | 63314983 | ENSBTAG00000000285 | protein_coding |
| *PHOX2B* | 6 | 60647109 | 60650018 | ENSBTAG00000044166 | protein_coding |
| *RBM47* | 6 | 59387155 | 59495126 | ENSBTAG00000002356 | protein_coding |
| *NSUN7* | 6 | 59663708 | 59710245 | ENSBTAG00000001572 | protein_coding |
| *APBB2* | 6 | 59714606 | 60095779 | ENSBTAG00000027569 | protein_coding |
| *UCHL1* | 6 | 60147289 | 60159346 | ENSBTAG00000005078 | protein_coding |
| *LIMCH1* | 6 | 60223942 | 60573879 | ENSBTAG00000010677 | protein_coding |
| *TMEM33* | 6 | 60814430 | 60835159 | ENSBTAG00000043958 | protein_coding |
| *SHISA3* | 6 | 61227698 | 61232482 | ENSBTAG00000049215 | protein_coding |
| *ATP8A1* | 6 | 61243693 | 61477908 | ENSBTAG00000011156 | protein_coding |
| *GRXCR1* | 6 | 61696746 | 61829436 | ENSBTAG00000032947 | protein_coding |
| *NWD2* | 6 | 56607403 | 56844579 | ENSBTAG00000001491 | protein_coding |
| *KCTD8* | 6 | 62834708 | 63097798 | ENSBTAG00000038659 | protein_coding |
| *GC* | 6 | 86963822 | 87007062 | ENSBTAG00000013718 | protein_coding |
| *ANKRD17* | 6 | 88187894 | 88355587 | ENSBTAG00000004912 | protein_coding |
| *ADAMTS3* | 6 | 87426966 | 87833898 | ENSBTAG00000006507 | protein_coding |
| *EPGN* | 6 | 89244813 | 89252276 | ENSBTAG00000004052 | protein_coding |
| *EREG* | 6 | 89306919 | 89325427 | ENSBTAG00000010273 | protein_coding |
| *AREG* | 6 | 89379645 | 89391792 | ENSBTAG00000018134 | protein_coding |
| *COX18* | 6 | 88173347 | 88184845 | ENSBTAG00000005394 | protein_coding |
| *AMTN* | 6 | 85903765 | 85917032 | ENSBTAG00000002928 | protein_coding |
| *AMBN* | 6 | 85968076 | 85979799 | ENSBTAG00000004793 | protein_coding |
| *ENAM* | 6 | 86007461 | 86021548 | ENSBTAG00000010346 | protein_coding |
| *JCHAIN* | 6 | 86032997 | 86071698 | ENSBTAG00000018531 | protein_coding |
| *RUFY3* | 6 | 86092616 | 86185301 | ENSBTAG00000016795 | protein_coding |
| *GRSF1* | 6 | 86196005 | 86250072 | ENSBTAG00000008577 | protein_coding |
| *MOB1B* | 6 | 86250301 | 86308361 | ENSBTAG00000016290 | protein_coding |
| *DCK* | 6 | 86319166 | 86345274 | ENSBTAG00000012397 | protein_coding |
| *SLC4A4* | 6 | 86449877 | 86809057 | ENSBTAG00000002348 | protein_coding |
| *SULT1B1* | 6 | 85185248 | 85216037 | ENSBTAG00000001249 | protein_coding |
| *SULT1E1* | 6 | 85309030 | 85365114 | ENSBTAG00000011952 | protein_coding |
| *CSN1S2* | 6 | 85530059 | 85548537 | ENSBTAG00000005005 | protein_coding |
| *ODAM* | 6 | 85594474 | 85603326 | ENSBTAG00000006810 | protein_coding |
| *CSN3* | 6 | 85644907 | 85658910 | ENSBTAG00000039787 | protein_coding |
| *CSN1S1* | 6 | 85411718 | 85429255 | ENSBTAG00000007695 | protein_coding |
| *CSN2* | 6 | 85449178 | 85457943 | ENSBTAG00000002632 | protein_coding |
| *HSTN* | 6 | 85459411 | 85472039 | ENSBTAG00000048250 | protein_coding |
| *CABS1* | 6 | 85734578 | 85736670 | ENSBTAG00000019849 | protein_coding |
| *UTP3* | 6 | 86071006 | 86074371 | ENSBTAG00000009310 | protein_coding |
| *MGC152010* | 6 | 84674164 | 84804636 | ENSBTAG00000053282 | protein_coding |
| *TECRL* | 6 | 79787721 | 79930156 | ENSBTAG00000024826 | protein_coding |
| *EPHA5* | 6 | 80836084 | 81240140 | ENSBTAG00000009438 | protein_coding |
| *TMPRSS11F* | 6 | 83749661 | 83844319 | ENSBTAG00000004412 | protein_coding |
| *TMPRSS11BNL* | 6 | 83890034 | 83911814 | ENSBTAG00000048377 | protein_coding |
| *TMPRSS11E* | 6 | 83956053 | 84012727 | ENSBTAG00000038520 | protein_coding |
| *YTHDC1* | 6 | 84101801 | 84139895 | ENSBTAG00000015572 | protein_coding |
| *UGT2B10* | 6 | 84443442 | 84464670 | ENSBTAG00000039991 | protein_coding |
| *ADGRL3* | 6 | 76931470 | 77652503 | ENSBTAG00000013918 | protein_coding |
| *COX7B2* | 6 | 65334067 | 65495543 | ENSBTAG00000050016 | protein_coding |
| *GABRA4* | 6 | 65496510 | 65573127 | ENSBTAG00000016645 | protein_coding |
| *GABRB1* | 6 | 65611892 | 66068619 | ENSBTAG00000017837 | protein_coding |
| *COMMD8* | 6 | 66095491 | 66110973 | ENSBTAG00000001348 | protein_coding |
| *ATP10D* | 6 | 66135885 | 66272135 | ENSBTAG00000000473 | protein_coding |
| *CORIN* | 6 | 66273891 | 66582006 | ENSBTAG00000002199 | protein_coding |
| *ARL9* | 6 | 71874850 | 71890264 | ENSBTAG00000010615 | protein_coding |
| *SPMAP2L* | 6 | 71894063 | 71935734 | ENSBTAG00000051380 | protein_coding |
| *NFXL1* | 6 | 66591304 | 66652258 | ENSBTAG00000002201 | protein_coding |
| *HOPX* | 6 | 71973666 | 71983218 | ENSBTAG00000002333 | protein_coding |
| *SPINK2B* | 6 | 72097152 | 72105211 | ENSBTAG00000032560 | protein_coding |
| *GNPDA2* | 6 | 63317754 | 63348386 | ENSBTAG00000000287 | protein_coding |
| *RESTB* | 6 | 72178448 | 72198298 | ENSBTAG00000011789 | protein_coding |
| *NOA1A* | 6 | 72349439 | 72361031 | ENSBTAG00000019362 | protein_coding |
| *POLR2B* | 6 | 72361714 | 72405885 | ENSBTAG00000019366 | protein_coding |
| *IGFBP7* | 6 | 72405520 | 72485427 | ENSBTAG00000019368 | protein_coding |
| *CNGA1* | 6 | 66668903 | 66706869 | ENSBTAG00000002205 | protein_coding |
| *NIPAL1* | 6 | 66717013 | 66745154 | ENSBTAG00000009423 | protein_coding |
| *TXK* | 6 | 66757241 | 66817720 | ENSBTAG00000005055 | protein_coding |
| *GABRG1* | 6 | 64584452 | 64674550 | ENSBTAG00000012296 | protein_coding |
| *TEC* | 6 | 66819043 | 66989142 | ENSBTAG00000005062 | protein_coding |
| *SLAIN2* | 6 | 67048701 | 67117951 | ENSBTAG00000021963 | protein_coding |
| *SLC10A4* | 6 | 67165376 | 67171369 | ENSBTAG00000004888 | protein_coding |
| *ZAR1* | 6 | 67172110 | 67187896 | ENSBTAG00000004886 | protein_coding |
| *FRYL* | 6 | 67179392 | 67377103 | ENSBTAG00000000137 | protein_coding |
| *OCIAD2* | 6 | 67528050 | 67539862 | ENSBTAG00000001839 | protein_coding |
| *FIP1L1* | 6 | 68940826 | 69005828 | ENSBTAG00000020653 | protein_coding |
| *LNX1* | 6 | 69006763 | 69133898 | ENSBTAG00000020658 | protein_coding |
| *GABRA2* | 6 | 64819338 | 64960787 | ENSBTAG00000011817 | protein_coding |
| *KDR* | 6 | 70567551 | 70612407 | ENSBTAG00000000782 | protein_coding |
| *CWH43* | 6 | 67633819 | 67703912 | ENSBTAG00000021347 | protein_coding |
| *DCUN1D4* | 6 | 67700449 | 67786559 | ENSBTAG00000001600 | protein_coding |
| *LRRC66* | 6 | 67853416 | 67878647 | ENSBTAG00000014599 | protein_coding |
| *SGCB* | 6 | 67878832 | 67906002 | ENSBTAG00000014601 | protein_coding |
| *SPATA18* | 6 | 67916506 | 67954443 | ENSBTAG00000018106 | protein_coding |
| *SRD5A3* | 6 | 70803119 | 70819349 | ENSBTAG00000014913 | protein_coding |
| *TMEM165* | 6 | 70838726 | 70865828 | ENSBTAG00000001269 | protein_coding |
| *CLOCK* | 6 | 70874084 | 70958310 | ENSBTAG00000044044 | protein_coding |
| *PDCL2* | 6 | 70988566 | 71024317 | ENSBTAG00000001772 | protein_coding |
| *EIF4E2* | 6 | 71005689 | 71129015 | ENSBTAG00000054466 | protein_coding |
| *EXOC1L* | 6 | 71230325 | 71247201 | ENSBTAG00000045602 | protein_coding |
| *EXOC1* | 6 | 71271841 | 71326113 | ENSBTAG00000032637 | protein_coding |
| *CHIC2* | 6 | 69523594 | 69596055 | ENSBTAG00000032660 | protein_coding |
| *GSX2* | 6 | 69629306 | 69631402 | ENSBTAG00000045812 | protein_coding |
| *PDGFRA* | 6 | 69723778 | 69771549 | ENSBTAG00000007173 | protein_coding |
| *KIT* | 6 | 70166692 | 70254044 | ENSBTAG00000002699 | protein_coding |
| *OCIAD1* | 6 | 67496284 | 67519280 | ENSBTAG00000010611 | protein_coding |
| *CEP135* | 6 | 71357447 | 71431834 | ENSBTAG00000009471 | protein_coding |
| *USP46* | 6 | 68303938 | 68378674 | ENSBTAG00000003443 | protein_coding |
| *RASL11B* | 6 | 68530424 | 68534897 | ENSBTAG00000020647 | protein_coding |
| *SCFD2* | 6 | 68538960 | 68936132 | ENSBTAG00000020648 | protein_coding |
| *CRACD* | 6 | 71604526 | 71731418 | ENSBTAG00000040398 | protein_coding |
| *AASDH* | 6 | 71742768 | 71772155 | ENSBTAG00000020583 | protein_coding |
| *PPAT* | 6 | 71782632 | 71821764 | ENSBTAG00000010571 | protein_coding |
| *SRP72* | 6 | 71846136 | 71871800 | ENSBTAG00000010593 | protein_coding |
| *U6* | 6 | 65987009 | 65987110 | ENSBTAG00000043245 | snRNA |
| *U2* | 6 | 67053157 | 67053258 | ENSBTAG00000053633 | snRNA |
| *U7* | 6 | 63816988 | 63817049 | ENSBTAG00000047521 | snRNA |
| *POU4F1* | 12 | 53744577 | 53746732 | ENSBTAG00000051184 | protein_coding |
| *OBI1* | 12 | 53756338 | 53802438 | ENSBTAG00000000869 | protein_coding |
| *EDNRB* | 12 | 53038377 | 53068132 | ENSBTAG00000005299 | protein_coding |
| *SLAIN1* | 12 | 52840174 | 52892757 | ENSBTAG00000003511 | protein_coding |
| *U6* | 12 | 53853456 | 53853562 | ENSBTAG00000043485 | snRNA |
| *bta-mir-2284s* | 12 | 52903108 | 52903175 | ENSBTAG00000044565 | miRNA |
| *PROK2* | 22 | 29859914 | 29883008 | ENSBTAG00000019330 | protein_coding |
| *CNTN6* | 22 | 24943366 | 25263794 | ENSBTAG00000003592 | protein_coding |
| *PDZRN3* | 22 | 28118157 | 28387641 | ENSBTAG00000018644 | protein_coding |
| *GPR27* | 22 | 29885605 | 29889517 | ENSBTAG00000058000 | protein_coding |
| *PSMD6* | 22 | 37420698 | 37439307 | ENSBTAG00000015112 | protein_coding |
| *ATXN7* | 22 | 37469886 | 37560061 | ENSBTAG00000011287 | protein_coding |
| *THOC7* | 22 | 37599652 | 37620599 | ENSBTAG00000032872 | protein_coding |
| *C22H3orf49* | 22 | 37620386 | 37631075 | ENSBTAG00000039133 | pseudogene |
| *PTPRG* | 22 | 39028806 | 39798723 | ENSBTAG00000021911 | protein_coding |
| *SUCLG2* | 22 | 33859296 | 34134865 | ENSBTAG00000009541 | protein_coding |
| *EOGT* | 22 | 32535096 | 32575454 | ENSBTAG00000022681 | protein_coding |
| *TAFA4* | 22 | 32581888 | 32790440 | ENSBTAG00000003110 | protein_coding |
| *TAFA1* | 22 | 32965288 | 33471946 | ENSBTAG00000019041 | protein_coding |
| *KBTBD8* | 22 | 34461519 | 34474228 | ENSBTAG00000011081 | protein_coding |
| *LRIG1* | 22 | 34953601 | 35067972 | ENSBTAG00000010360 | protein_coding |
| *SLC25A26* | 22 | 35068139 | 35215058 | ENSBTAG00000037972 | protein_coding |
| *MDFIC2* | 22 | 31286348 | 31399636 | ENSBTAG00000064171 | protein_coding |
| *MITF* | 22 | 31616846 | 31857969 | ENSBTAG00000006679 | protein_coding |
| *MAGI1* | 22 | 35381753 | 36021215 | ENSBTAG00000010581 | protein_coding |
| *RASSF1* | 22 | 50006866 | 50015924 | ENSBTAG00000020963 | protein_coding |
| *TUSC2* | 22 | 50016675 | 50022052 | ENSBTAG00000058889 | protein_coding |
| *HYAL2* | 22 | 50021694 | 50029994 | ENSBTAG00000000484 | protein_coding |
| *HYAL1* | 22 | 50030328 | 50033370 | ENSBTAG00000000483 | protein_coding |
| *IFRD2* | 22 | 50063348 | 50066774 | ENSBTAG00000000480 | protein_coding |
| *SEMA3B* | 22 | 50084354 | 50091200 | ENSBTAG00000010138 | protein_coding |
| *GNAI2* | 22 | 50099986 | 50120522 | ENSBTAG00000020645 | protein_coding |
| *SLC38A3* | 22 | 50131902 | 50146657 | ENSBTAG00000008509 | protein_coding |
| *GNAT1* | 22 | 50152601 | 50161258 | ENSBTAG00000018020 | protein_coding |
| *SNORD22* | 22 | 34577370 | 34577495 | ENSBTAG00000042763 | snoRNA |
| *U6* | 22 | 34637832 | 34637938 | ENSBTAG00000045749 | snRNA |
| *bta-mir-2369* | 22 | 39677352 | 39677421 | ENSBTAG00000044501 | miRNA |
| *CACNA2D2* | 22 | 49846519 | 49985938 | ENSBTAG00000009489 | protein_coding |
| *XCR1* | 22 | 53300562 | 53324371 | ENSBTAG00000063018 | protein_coding |
| *FYCO1* | 22 | 53325781 | 53401616 | ENSBTAG00000015698 | protein_coding |
| *CXCR6* | 22 | 53366881 | 53379701 | ENSBTAG00000015708 | protein_coding |
| *TMEM115* | 22 | 49989078 | 49994447 | ENSBTAG00000058435 | protein_coding |
| *CYB561D2* | 22 | 49994640 | 49997688 | ENSBTAG00000019163 | protein_coding |
| *NPRL2* | 22 | 49997691 | 50001018 | ENSBTAG00000019161 | protein_coding |
| *ZMYND10* | 22 | 50002503 | 50006653 | ENSBTAG00000020965 | protein_coding |
| *HYAL3* | 22 | 50033536 | 50039609 | ENSBTAG00000052388 | protein_coding |
| *LSMEM2* | 22 | 50067243 | 50070107 | ENSBTAG00000000478 | protein_coding |

lncRNA: long non-coding RNA; miRNA: microRNA; snoRNA: small nucleolar RNA; snRNA: small nuclear RNA.

**Table 3.** Gene Ontology terms for the genes annotated for depigmentation trait

| Functional terms | Source | Description of function | Genes |
| --- | --- | --- | --- |
| GO:0009127 | GO:BP | purine nucleoside monophosphate biosynthetic process | ENSBTAG00000012397,ENSBTAG00000010571,ENSBTAG00000060590,ENSBTAG00000010577 |
| GO:0006189 | GO:BP | 'de novo' IMP biosynthetic process | ENSBTAG00000010571,ENSBTAG00000060590,ENSBTAG00000010577 |
| GO:0007214 | GO:BP | gamma-aminobutyric acid signaling pathway | ENSBTAG00000016645,ENSBTAG00000017837,ENSBTAG00000011817,ENSBTAG00000020645 |
| GO:0071493 | GO:BP | cellular response to UV-B | ENSBTAG00000000484,ENSBTAG00000000483,ENSBTAG00000052388 |
| GO:0051145 | GO:BP | smooth muscle cell differentiation | ENSBTAG00000004912,ENSBTAG00000010273,ENSBTAG00000014601,ENSBTAG00000002699,ENSBTAG00000005299 |
| GO:1902711 | GO:CC | GABA-A receptor complex | ENSBTAG00000016645,ENSBTAG00000017837,ENSBTAG00000011817 |
| GO:1902710 | GO:CC | GABA receptor complex | ENSBTAG00000016645,ENSBTAG00000017837,ENSBTAG00000011817 |
| GO:0015020 | GO:MF | glucuronosyltransferase activity | ENSBTAG00000059934,ENSBTAG00000060805,ENSBTAG00000053565,ENSBTAG00000053282,ENSBTAG00000039991,ENSBTAG00000058539 |
| GO:0008194 | GO:MF | UDP-glycosyltransferase activity | ENSBTAG00000059934,ENSBTAG00000060805,ENSBTAG00000053565,ENSBTAG00000053282,ENSBTAG00000039991,ENSBTAG00000058539,ENSBTAG00000022681,ENSBTAG00000000483 |
| GO:0019957 | GO:MF | C-C chemokine binding | ENSBTAG00000019428,ENSBTAG00000031355,ENSBTAG00000063018,ENSBTAG00000015708 |
| GO:0001637 | GO:MF | G protein-coupled chemoattractant receptor activity | ENSBTAG00000019428,ENSBTAG00000031355,ENSBTAG00000063018,ENSBTAG00000015708 |
| GO:0004950 | GO:MF | chemokine receptor activity | ENSBTAG00000019428,ENSBTAG00000031355,ENSBTAG00000063018,ENSBTAG00000015708 |
| GO:0016493 | GO:MF | C-C chemokine receptor activity | ENSBTAG00000019428,ENSBTAG00000031355,ENSBTAG00000063018,ENSBTAG00000015708 |
| GO:0004415 | GO:MF | hyalurononglucosaminidase activity | ENSBTAG00000000484,ENSBTAG00000000483,ENSBTAG00000052388 |
| GO:0004639 | GO:MF | phosphoribosylaminoimidazolesuccinocarboxamide synthase activity | ENSBTAG00000060590,ENSBTAG00000010577 |
| GO:0004638 | GO:MF | phosphoribosylaminoimidazole carboxylase activity | ENSBTAG00000060590,ENSBTAG00000010577 |
| GO:0035717 | GO:MF | chemokine (C-C motif) ligand 7 binding | ENSBTAG00000019428,ENSBTAG00000031355 |
| GO:0033906 | GO:MF | hyaluronoglucuronidase activity | ENSBTAG00000000484,ENSBTAG00000052388 |
| GO:0016758 | GO:MF | hexosyltransferase activity | ENSBTAG00000059934,ENSBTAG00000060805,ENSBTAG00000053565,ENSBTAG00000053282,ENSBTAG00000039991,ENSBTAG00000058539,ENSBTAG00000022681,ENSBTAG00000000483 |
| GO:0071791 | GO:MF | chemokine (C-C motif) ligand 5 binding | ENSBTAG00000019428,ENSBTAG00000031355 |
| GO:0019956 | GO:MF | chemokine binding | ENSBTAG00000019428,ENSBTAG00000031355,ENSBTAG00000063018,ENSBTAG00000015708 |
| GO:0030297 | GO:MF | transmembrane receptor protein tyrosine kinase activator activity | ENSBTAG00000004052,ENSBTAG00000010273,ENSBTAG00000018134 |
| GO:0016757 | GO:MF | glycosyltransferase activity | ENSBTAG00000059934,ENSBTAG00000060805,ENSBTAG00000053565,ENSBTAG00000053282,ENSBTAG00000039991,ENSBTAG00000058539,ENSBTAG00000010571,ENSBTAG00000022681,ENSBTAG00000000483 |
| GO:0030021 | GO:MF | extracellular matrix structural constituent conferring compression resistance | ENSBTAG00000004793,ENSBTAG00000010346 |
| GO:0030345 | GO:MF | structural constituent of tooth enamel | ENSBTAG00000004793,ENSBTAG00000010346 |
| GO:0004713 | GO:MF | protein tyrosine kinase activity | ENSBTAG00000009438,ENSBTAG00000005055,ENSBTAG00000005062,ENSBTAG00000000782,ENSBTAG00000007173,ENSBTAG00000002699 |
| GO:0015929 | GO:MF | hexosaminidase activity | ENSBTAG00000000484,ENSBTAG00000000483,ENSBTAG00000052388 |
| GO:0038085 | GO:MF | vascular endothelial growth factor binding | ENSBTAG00000000782,ENSBTAG00000007173 |
| GO:0019955 | GO:MF | cytokine binding | ENSBTAG00000002699,ENSBTAG00000000484,ENSBTAG00000019428,ENSBTAG00000031355,ENSBTAG00000063018,ENSBTAG00000015708 |
| GO:0004062 | GO:MF | aryl sulfotransferase activity | ENSBTAG00000001249,ENSBTAG00000038214 |
| GO:0004714 | GO:MF | transmembrane receptor protein tyrosine kinase activity | ENSBTAG00000009438,ENSBTAG00000000782,ENSBTAG00000007173,ENSBTAG00000002699 |
| GO:0030296 | GO:MF | protein tyrosine kinase activator activity | ENSBTAG00000004052,ENSBTAG00000010273,ENSBTAG00000018134 |
| GO:0005021 | GO:MF | vascular endothelial growth factor receptor activity | ENSBTAG00000000782,ENSBTAG00000007173 |
| GO:0005154 | GO:MF | epidermal growth factor receptor binding | ENSBTAG00000004052,ENSBTAG00000010273,ENSBTAG00000018134 |
| GO:0019199 | GO:MF | transmembrane receptor protein kinase activity | ENSBTAG00000009438,ENSBTAG00000000782,ENSBTAG00000007173,ENSBTAG00000002699 |
| GO:0022851 | GO:MF | GABA-gated chloride ion channel activity | ENSBTAG00000017837,ENSBTAG00000011817 |
| KEGG:00140 | KEGG | Steroid hormone biosynthesis | ENSBTAG00000059934,ENSBTAG00000053565,ENSBTAG00000011952,ENSBTAG00000053282,ENSBTAG00000064079,ENSBTAG00000039991,ENSBTAG00000058539,ENSBTAG00000014913 |
| KEGG:00053 | KEGG | Ascorbate and aldarate metabolism | ENSBTAG00000059934,ENSBTAG00000053565,ENSBTAG00000053282,ENSBTAG00000064079,ENSBTAG00000039991,ENSBTAG00000058539 |
| KEGG:00040 | KEGG | Pentose and glucuronate interconversions | ENSBTAG00000059934,ENSBTAG00000053565,ENSBTAG00000053282,ENSBTAG00000064079,ENSBTAG00000039991,ENSBTAG00000058539 |
| KEGG:00860 | KEGG | Porphyrin metabolism | ENSBTAG00000059934,ENSBTAG00000053565,ENSBTAG00000053282,ENSBTAG00000064079,ENSBTAG00000039991,ENSBTAG00000058539 |
| KEGG:00982 | KEGG | Drug metabolism - cytochrome P450 | ENSBTAG00000059934,ENSBTAG00000053565,ENSBTAG00000053282,ENSBTAG00000064079,ENSBTAG00000039991,ENSBTAG00000058539 |
| KEGG:05204 | KEGG | Chemical carcinogenesis - DNA adducts | ENSBTAG00000059934,ENSBTAG00000053565,ENSBTAG00000053282,ENSBTAG00000064079,ENSBTAG00000039991,ENSBTAG00000058539 |
| KEGG:04976 | KEGG | Bile secretion | ENSBTAG00000002348,ENSBTAG00000059934,ENSBTAG00000053565,ENSBTAG00000053282,ENSBTAG00000064079,ENSBTAG00000039991,ENSBTAG00000058539 |
| KEGG:00980 | KEGG | Metabolism of xenobiotics by cytochrome P450 | ENSBTAG00000059934,ENSBTAG00000053565,ENSBTAG00000053282,ENSBTAG00000064079,ENSBTAG00000039991,ENSBTAG00000058539 |
| KEGG:00830 | KEGG | Retinol metabolism | ENSBTAG00000059934,ENSBTAG00000053565,ENSBTAG00000053282,ENSBTAG00000064079,ENSBTAG00000039991,ENSBTAG00000058539 |
| KEGG:00983 | KEGG | Drug metabolism - other enzymes | ENSBTAG00000059934,ENSBTAG00000053565,ENSBTAG00000053282,ENSBTAG00000064079,ENSBTAG00000039991,ENSBTAG00000058539 |
| KEGG:04727 | KEGG | GABAergic synapse | ENSBTAG00000016645,ENSBTAG00000017837,ENSBTAG00000012296,ENSBTAG00000011817,ENSBTAG00000020645,ENSBTAG00000008509 |
| KEGG:05033 | KEGG | Nicotine addiction | ENSBTAG00000016645,ENSBTAG00000017837,ENSBTAG00000012296,ENSBTAG00000011817 |
| KEGG:05032 | KEGG | Morphine addiction | ENSBTAG00000016645,ENSBTAG00000017837,ENSBTAG00000012296,ENSBTAG00000011817,ENSBTAG00000020645 |
| KEGG:05207 | KEGG | Chemical carcinogenesis - receptor activation | ENSBTAG00000059934,ENSBTAG00000053565,ENSBTAG00000053282,ENSBTAG00000064079,ENSBTAG00000039991,ENSBTAG00000058539,ENSBTAG00000020645 |
| KEGG:01240 | KEGG | Biosynthesis of cofactors | ENSBTAG00000059934,ENSBTAG00000053565,ENSBTAG00000053282,ENSBTAG00000064079,ENSBTAG00000039991,ENSBTAG00000058539 |
| KEGG:04723 | KEGG | Retrograde endocannabinoid signaling | ENSBTAG00000016645,ENSBTAG00000017837,ENSBTAG00000012296,ENSBTAG00000011817,ENSBTAG00000020645 |
| KEGG:04916 | KEGG | Melanogenesis | ENSBTAG00000002699,ENSBTAG00000005299,ENSBTAG00000006679,ENSBTAG00000020645 |

GO: Gene Ontology; BP: Biological Process; CC: Cellular Component; MF: Molecular Function; KEGG: Kyoto Encyclopedia of Genes and Genomes

**Table 4.** Positional candidate genes located near the regions identified in this study after conditional GWAS

| Gene | Chromosome | Genomic Region (Start – End) | | gene_id | gene_biotype |
| --- | --- | --- | --- | --- | --- |
|  | 6 | 49373331 | 49388249 | ENSBTAG00000071567 | lncRNA |
| *EPHA5* | 6 | 80836084 | 81240140 | ENSBTAG00000009438 | protein_coding |
|  | 6 | 84381930 | 84402980 | ENSBTAG00000064079 | protein_coding |
| *CSN1S1* | 6 | 85411574 | 85429255 | ENSBTAG00000007695 | protein_coding |
| *UGT2B10* | 6 | 84443442 | 84464670 | ENSBTAG00000039991 | protein_coding |
| *CSN2* | 6 | 85449178 | 85457943 | ENSBTAG00000002632 | protein_coding |
|  | 6 | 85467458 | 85472039 | ENSBTAG00000048250 | protein_coding |
|  | 6 | 84473140 | 84513202 | ENSBTAG00000059934 | protein_coding |
|  | 6 | 85497254 | 85506606 | ENSBTAG00000060764 | protein_coding |
| *SULT1B1* | 6 | 85185248 | 85216037 | ENSBTAG00000001249 | protein_coding |
| *SULT1D1* | 6 | 85256039 | 85281712 | ENSBTAG00000038214 | protein_coding |
| *SULT1E1* | 6 | 85309030 | 85365114 | ENSBTAG00000011952 | protein_coding |
| *CSN1S2* | 6 | 85530037 | 85548537 | ENSBTAG00000005005 | protein_coding |
|  | 6 | 83687443 | 83729169 | ENSBTAG00000025920 | protein_coding |
| *TMPRSS11F* | 6 | 83749661 | 83844319 | ENSBTAG00000004412 | protein_coding |
| *TMPRSS11BNL* | 6 | 83890034 | 83911814 | ENSBTAG00000048377 | protein_coding |
|  | 6 | 83916884 | 83918314 | ENSBTAG00000032360 | protein_coding |
|  | 6 | 83921912 | 83936866 | ENSBTAG00000064912 | pseudogene |
| *TMPRSS11E* | 6 | 83956053 | 84012727 | ENSBTAG00000038520 | protein_coding |
| *YTHDC1* | 6 | 84101658 | 84139900 | ENSBTAG00000015572 | protein_coding |
|  | 6 | 79181662 | 79182972 | ENSBTAG00000054580 | protein_coding |
| *ADGRL3* | 6 | 76931470 | 77652241 | ENSBTAG00000013918 | protein_coding |
| *TECRL* | 6 | 79787721 | 79930156 | ENSBTAG00000024826 | protein_coding |
|  | 6 | 76699283 | 76716492 | ENSBTAG00000057966 | lncRNA |
|  | 6 | 76807179 | 76840376 | ENSBTAG00000064817 | lncRNA |
|  | 6 | 77653565 | 77655878 | ENSBTAG00000075491 | lncRNA |
|  | 6 | 77657846 | 77659553 | ENSBTAG00000078149 | lncRNA |
|  | 6 | 81240451 | 81288902 | ENSBTAG00000055547 | lncRNA |
|  | 6 | 84433340 | 84435470 | ENSBTAG00000060336 | lncRNA |
|  | 6 | 84441063 | 84443356 | ENSBTAG00000075612 | lncRNA |
|  | 6 | 85173542 | 85183607 | ENSBTAG00000061770 | lncRNA |
|  | 6 | 85224760 | 85239161 | ENSBTAG00000078382 | lncRNA |
|  | 6 | 85230496 | 85233242 | ENSBTAG00000072301 | lncRNA |
|  | 6 | 85458569 | 85461210 | ENSBTAG00000072804 | lncRNA |
|  | 6 | 85549650 | 85567175 | ENSBTAG00000072829 | lncRNA |
|  | 6 | 81258101 | 81390388 | ENSBTAG00000078584 | lncRNA |
|  | 6 | 84636600 | 84662087 | ENSBTAG00000062982 | lncRNA |
| *U6* | 6 | 84058604 | 84058705 | ENSBTAG00000044396 | snRNA |
|  | 6 | 77012506 | 77012577 | ENSBTAG00000073146 | miRNA |
|  | 6 | 81283203 | 81283261 | ENSBTAG00000076820 | miRNA |
|  | 6 | 84295812 | 84360717 | ENSBTAG00000058539 | protein_coding |
| *MGC152010* | 6 | 84674164 | 84847755 | ENSBTAG00000053282 | protein_coding |
| *NFXL1* | 6 | 66591304 | 66652258 | ENSBTAG00000002201 | protein_coding |
| *SPINK2B* | 6 | 72097152 | 72104516 | ENSBTAG00000032560 | protein_coding |
| *OCIAD1* | 6 | 67496261 | 67519221 | ENSBTAG00000010611 | protein_coding |
| *KDR* | 6 | 70567551 | 70612407 | ENSBTAG00000000782 | protein_coding |
| *CNGA1* | 6 | 66668903 | 66706869 | ENSBTAG00000002205 | protein_coding |
| *NIPAL1* | 6 | 66717024 | 66747656 | ENSBTAG00000009423 | protein_coding |
| *TXK* | 6 | 66757241 | 66817720 | ENSBTAG00000005055 | protein_coding |
| *GABRA4* | 6 | 65496510 | 65573127 | ENSBTAG00000016645 | protein_coding |
| *TEC* | 6 | 66819043 | 66989142 | ENSBTAG00000005062 | protein_coding |
| *SLAIN2* | 6 | 67048630 | 67120454 | ENSBTAG00000021963 | protein_coding |
| *SLC10A4* | 6 | 67165376 | 67171369 | ENSBTAG00000004888 | protein_coding |
| *ZAR1* | 6 | 67172110 | 67176434 | ENSBTAG00000004886 | protein_coding |
| *FRYL* | 6 | 67179392 | 67377103 | ENSBTAG00000000137 | protein_coding |
| *SRD5A3* | 6 | 70803119 | 70821248 | ENSBTAG00000014913 | protein_coding |
| *TMEM165* | 6 | 70838664 | 70865828 | ENSBTAG00000001269 | protein_coding |
| *CLOCK* | 6 | 70874084 | 70958310 | ENSBTAG00000044044 | protein_coding |
| *OCIAD2* | 6 | 67528050 | 67539862 | ENSBTAG00000001839 | protein_coding |
| *PDCL2* | 6 | 70988566 | 71024317 | ENSBTAG00000001772 | protein_coding |
| *EIF4E2* | 6 | 71005689 | 71129015 | ENSBTAG00000054466 | protein_coding |
| *NMU* | 6 | 71033006 | 71061423 | ENSBTAG00000074433 | protein_coding |
| *EXOC1L* | 6 | 71230325 | 71247201 | ENSBTAG00000045602 | protein_coding |
| *GABRB1* | 6 | 65611637 | 66068619 | ENSBTAG00000017837 | protein_coding |
| *EXOC1* | 6 | 71271833 | 71326393 | ENSBTAG00000032637 | protein_coding |
| *CEP135* | 6 | 71357336 | 71433278 | ENSBTAG00000009471 | protein_coding |
| *RESTB* | 6 | 72178448 | 72199319 | ENSBTAG00000011789 | protein_coding |
| *NOA1A* | 6 | 72349439 | 72361031 | ENSBTAG00000019362 | protein_coding |
| *POLR2B* | 6 | 72361710 | 72405885 | ENSBTAG00000019366 | protein_coding |
|  | 6 | 72362614 | 72363420 | ENSBTAG00000051484 | protein_coding |
| *IGFBP7* | 6 | 72405520 | 72485427 | ENSBTAG00000019368 | protein_coding |
| *KIT* | 6 | 70166682 | 70254046 | ENSBTAG00000002699 | protein_coding |
| *COMMD8* | 6 | 66095491 | 66110973 | ENSBTAG00000001348 | protein_coding |
| *CRACD* | 6 | 71456715 | 71731124 | ENSBTAG00000040398 | protein_coding |
| *AASDH* | 6 | 71742768 | 71772155 | ENSBTAG00000020583 | protein_coding |
| *PPAT* | 6 | 71782632 | 71821764 | ENSBTAG00000010571 | protein_coding |
|  | 6 | 71796663 | 71844034 | ENSBTAG00000010577 | protein_coding |
| *SRP72* | 6 | 71846130 | 71872375 | ENSBTAG00000010593 | protein_coding |
| *ARL9* | 6 | 71871296 | 71890028 | ENSBTAG00000010615 | protein_coding |
| *SPMAP2L* | 6 | 71894055 | 71935729 | ENSBTAG00000051380 | protein_coding |
| *CWH43* | 6 | 67633819 | 67703912 | ENSBTAG00000021347 | protein_coding |
| *DCUN1D4* | 6 | 67706962 | 67788864 | ENSBTAG00000001600 | protein_coding |
| *LRRC66* | 6 | 67853416 | 67878647 | ENSBTAG00000014599 | protein_coding |
| *SGCB* | 6 | 67878832 | 67906002 | ENSBTAG00000014601 | protein_coding |
| *SPATA18* | 6 | 67916302 | 67960213 | ENSBTAG00000018106 | protein_coding |
| *ATP10D* | 6 | 66135885 | 66272136 | ENSBTAG00000000473 | protein_coding |
| *CORIN* | 6 | 66273891 | 66582006 | ENSBTAG00000002199 | protein_coding |
|  | 6 | 66310268 | 66312380 | ENSBTAG00000072808 | protein_coding |
|  | 6 | 69402213 | 69463110 | ENSBTAG00000059729 | protein_coding |
| *HOPX* | 6 | 71973666 | 71983218 | ENSBTAG00000002333 | protein_coding |
| *CHIC2* | 6 | 69523594 | 69596055 | ENSBTAG00000032660 | protein_coding |
| *GSX2* | 6 | 69629123 | 69631474 | ENSBTAG00000045812 | protein_coding |
| *PDGFRA* | 6 | 69724630 | 69771544 | ENSBTAG00000007173 | protein_coding |
| *USP46* | 6 | 68303938 | 68378674 | ENSBTAG00000003443 | protein_coding |
| *RASL11B* | 6 | 68530424 | 68534897 | ENSBTAG00000020647 | protein_coding |
| *SCFD2* | 6 | 68538960 | 68936132 | ENSBTAG00000020648 | protein_coding |
| *FIP1L1* | 6 | 68940909 | 69008886 | ENSBTAG00000020653 | protein_coding |
| *LNX1* | 6 | 69006763 | 69133898 | ENSBTAG00000020658 | protein_coding |
| *GABRG1* | 6 | 64584452 | 64674550 | ENSBTAG00000012296 | protein_coding |
| *GABRA2* | 6 | 64819338 | 64960787 | ENSBTAG00000011817 | protein_coding |
| *COX7B2* | 6 | 65334350 | 65334589 | ENSBTAG00000050016 | protein_coding |
|  | 6 | 65420877 | 65421244 | ENSBTAG00000074758 | protein_coding |
|  | 6 | 71964073 | 71964546 | ENSBTAG00000075619 | protein_coding |
|  | 6 | 64814826 | 64817514 | ENSBTAG00000076926 | lncRNA |
|  | 6 | 64814829 | 64818263 | ENSBTAG00000075218 | lncRNA |
|  | 6 | 65406820 | 65411036 | ENSBTAG00000074402 | lncRNA |
|  | 6 | 65488155 | 65490008 | ENSBTAG00000073566 | lncRNA |
|  | 6 | 65492148 | 65493666 | ENSBTAG00000070539 | lncRNA |
|  | 6 | 66072453 | 66076115 | ENSBTAG00000078426 | lncRNA |
|  | 6 | 66714974 | 66717002 | ENSBTAG00000060489 | lncRNA |
|  | 6 | 66753900 | 66756632 | ENSBTAG00000066291 | lncRNA |
|  | 6 | 66989859 | 67005306 | ENSBTAG00000057787 | lncRNA |
|  | 6 | 67122261 | 67124442 | ENSBTAG00000060289 | lncRNA |
|  | 6 | 67128206 | 67129602 | ENSBTAG00000060841 | lncRNA |
|  | 6 | 67164037 | 67165237 | ENSBTAG00000059749 | lncRNA |
|  | 6 | 67388632 | 67431497 | ENSBTAG00000066111 | lncRNA |
|  | 6 | 67411655 | 67447781 | ENSBTAG00000057806 | lncRNA |
|  | 6 | 67433126 | 67448502 | ENSBTAG00000061642 | lncRNA |
|  | 6 | 67447908 | 67449154 | ENSBTAG00000077431 | lncRNA |
|  | 6 | 67481857 | 67485471 | ENSBTAG00000075312 | lncRNA |
|  | 6 | 67810104 | 67816201 | ENSBTAG00000063093 | lncRNA |
|  | 6 | 68524546 | 68527238 | ENSBTAG00000057770 | lncRNA |
|  | 6 | 69157723 | 69162086 | ENSBTAG00000068786 | lncRNA |
|  | 6 | 69244647 | 69249405 | ENSBTAG00000066737 | lncRNA |
|  | 6 | 69245220 | 69247299 | ENSBTAG00000068721 | lncRNA |
|  | 6 | 69517892 | 69521010 | ENSBTAG00000078640 | lncRNA |
|  | 6 | 69692001 | 69722083 | ENSBTAG00000058666 | lncRNA |
|  | 6 | 70565018 | 70566426 | ENSBTAG00000067038 | lncRNA |
|  | 6 | 70833830 | 70838165 | ENSBTAG00000073849 | lncRNA |
|  | 6 | 71137026 | 71155517 | ENSBTAG00000077145 | lncRNA |
|  | 6 | 71155262 | 71156516 | ENSBTAG00000078537 | lncRNA |
|  | 6 | 71268913 | 71271778 | ENSBTAG00000065353 | lncRNA |
|  | 6 | 71353717 | 71356900 | ENSBTAG00000060009 | lncRNA |
|  | 6 | 71354389 | 71356673 | ENSBTAG00000056238 | lncRNA |
|  | 6 | 71777185 | 71778261 | ENSBTAG00000066131 | lncRNA |
|  | 6 | 71780603 | 71782102 | ENSBTAG00000071396 | lncRNA |
|  | 6 | 72002730 | 72005978 | ENSBTAG00000056696 | lncRNA |
|  | 6 | 72113387 | 72116053 | ENSBTAG00000062483 | lncRNA |
|  | 6 | 69171339 | 69205252 | ENSBTAG00000062542 | lncRNA |
|  | 6 | 70614576 | 70713391 | ENSBTAG00000065274 | lncRNA |
|  | 6 | 70973551 | 70978532 | ENSBTAG00000067426 | lncRNA |
|  | 6 | 71138689 | 71201153 | ENSBTAG00000070286 | lncRNA |
| *U6* | 6 | 71845309 | 71845369 | ENSBTAG00000044539 | snRNA |
|  | 6 | 72081095 | 72081221 | ENSBTAG00000042899 | snRNA |
| *U2* | 6 | 67053157 | 67053258 | ENSBTAG00000053633 | snRNA |
| *U6* | 6 | 69877227 | 69877333 | ENSBTAG00000072816 | snRNA |
| *U6* | 6 | 70557421 | 70557527 | ENSBTAG00000076883 | snRNA |
| *U6* | 6 | 70521225 | 70521316 | ENSBTAG00000045439 | snRNA |
| *U6* | 6 | 69954510 | 69954616 | ENSBTAG00000076226 | snRNA |
| *U6* | 6 | 65987009 | 65987110 | ENSBTAG00000043245 | snRNA |
| *Y_RNA* | 6 | 75204362 | 75204457 | ENSBTAG00000043492 | Y_RNA |
|  | 6 | 68256017 | 68256044 | ENSBTAG00000070593 | miRNA |
|  | 6 | 69912429 | 69912493 | ENSBTAG00000071597 | miRNA |
|  | 6 | 69748104 | 69748135 | ENSBTAG00000077753 | miRNA |
| *RESTA* | 6 | 72320467 | 72335491 | ENSBTAG00000068505 | protein_coding |
| *RBM47* | 6 | 59387155 | 59495126 | ENSBTAG00000002356 | protein_coding |
| *NSUN7* | 6 | 59663448 | 59709986 | ENSBTAG00000001572 | protein_coding |
| *APBB2* | 6 | 59714606 | 60095779 | ENSBTAG00000027569 | protein_coding |
| *UCHL1* | 6 | 60147289 | 60159346 | ENSBTAG00000005078 | protein_coding |
| *LIMCH1* | 6 | 60224047 | 60575764 | ENSBTAG00000010677 | protein_coding |
| *PHOX2B* | 6 | 60647109 | 60650018 | ENSBTAG00000044166 | protein_coding |
| *NWD2* | 6 | 56607451 | 56844579 | ENSBTAG00000001491 | protein_coding |
| *TMEM33* | 6 | 60814866 | 60835159 | ENSBTAG00000043958 | protein_coding |
| *ARAP2* | 6 | 55444521 | 55641486 | ENSBTAG00000039922 | protein_coding |
| *DTHD1* | 6 | 55680843 | 55762587 | ENSBTAG00000009833 | protein_coding |
|  | 6 | 55747978 | 55750303 | ENSBTAG00000079009 | protein_coding |
| *SHISA3* | 6 | 61227698 | 61232482 | ENSBTAG00000049215 | protein_coding |
| *ATP8A1* | 6 | 61243693 | 61477856 | ENSBTAG00000011156 | protein_coding |
| *GRXCR1* | 6 | 61696746 | 61829436 | ENSBTAG00000032947 | protein_coding |
| *KCTD8* | 6 | 62834708 | 63097798 | ENSBTAG00000038659 | protein_coding |
| *YIPF7* | 6 | 63238860 | 63290332 | ENSBTAG00000001603 | protein_coding |
| *GUF1* | 6 | 63290166 | 63317737 | ENSBTAG00000000285 | protein_coding |
| *GNPDA2* | 6 | 63317754 | 63348386 | ENSBTAG00000000287 | protein_coding |
|  | 6 | 55644217 | 55646527 | ENSBTAG00000061912 | lncRNA |
|  | 6 | 56586665 | 56589818 | ENSBTAG00000068440 | lncRNA |
|  | 6 | 59338905 | 59343431 | ENSBTAG00000065836 | lncRNA |
|  | 6 | 59356337 | 59378106 | ENSBTAG00000063896 | lncRNA |
|  | 6 | 59562628 | 59584688 | ENSBTAG00000064140 | lncRNA |
|  | 6 | 59608689 | 59611486 | ENSBTAG00000062472 | lncRNA |
|  | 6 | 60099433 | 60124492 | ENSBTAG00000078806 | lncRNA |
|  | 6 | 60163860 | 60171872 | ENSBTAG00000075749 | lncRNA |
|  | 6 | 60199290 | 60201292 | ENSBTAG00000078607 | lncRNA |
|  | 6 | 61050195 | 61119715 | ENSBTAG00000069527 | lncRNA |
|  | 6 | 61102376 | 61103745 | ENSBTAG00000071752 | lncRNA |
|  | 6 | 61131420 | 61135047 | ENSBTAG00000064806 | lncRNA |
|  | 6 | 61167703 | 61174105 | ENSBTAG00000069038 | lncRNA |
|  | 6 | 61479620 | 61493469 | ENSBTAG00000063795 | lncRNA |
|  | 6 | 61587790 | 61589753 | ENSBTAG00000077803 | lncRNA |
|  | 6 | 63528958 | 63531802 | ENSBTAG00000071729 | lncRNA |
|  | 6 | 59586475 | 59613987 | ENSBTAG00000069711 | lncRNA |
|  | 6 | 60101077 | 60105743 | ENSBTAG00000074115 | lncRNA |
|  | 6 | 60167194 | 60174688 | ENSBTAG00000068437 | lncRNA |
|  | 6 | 60183372 | 60187623 | ENSBTAG00000073643 | lncRNA |
|  | 6 | 60179036 | 60195216 | ENSBTAG00000056559 | lncRNA |
|  | 6 | 61478439 | 61592222 | ENSBTAG00000058079 | lncRNA |
|  | 6 | 63508649 | 63806680 | ENSBTAG00000077379 | lncRNA |
|  | 6 | 63939223 | 64057964 | ENSBTAG00000078988 | lncRNA |
|  | 6 | 64054967 | 64099887 | ENSBTAG00000075736 | lncRNA |
| *U7* | 6 | 63816988 | 63817049 | ENSBTAG00000047521 | snRNA |
|  | 6 | 63097081 | 63097180 | ENSBTAG00000070430 | miRNA |
| *ANKRD17* | 6 | 88187894 | 88355587 | ENSBTAG00000004912 | protein_coding |
| *EPGN* | 6 | 89244813 | 89252276 | ENSBTAG00000004052 | protein_coding |
| *EREG* | 6 | 89306948 | 89327099 | ENSBTAG00000010273 | protein_coding |
| *AREG* | 6 | 89379645 | 89391878 | ENSBTAG00000018134 | protein_coding |
| *ODAM* | 6 | 85594474 | 85603326 | ENSBTAG00000006810 | protein_coding |
| *CSN3* | 6 | 85644907 | 85670255 | ENSBTAG00000039787 | protein_coding |
| *CABS1* | 6 | 85734578 | 85736670 | ENSBTAG00000019849 | protein_coding |
|  | 6 | 85775386 | 85780140 | ENSBTAG00000061217 | protein_coding |
| *AMTN* | 6 | 85903765 | 85917032 | ENSBTAG00000002928 | protein_coding |
| *AMBN* | 6 | 85968076 | 85979799 | ENSBTAG00000004793 | protein_coding |
| *ENAM* | 6 | 86007461 | 86023449 | ENSBTAG00000010346 | protein_coding |
| *JCHAIN* | 6 | 86032997 | 86071595 | ENSBTAG00000018531 | protein_coding |
| *RUFY3* | 6 | 86092758 | 86185579 | ENSBTAG00000016795 | protein_coding |
| *DCK* | 6 | 86319009 | 86345273 | ENSBTAG00000012397 | protein_coding |
| *GRSF1* | 6 | 86196005 | 86250072 | ENSBTAG00000008577 | protein_coding |
| *MOB1B* | 6 | 86250279 | 86308361 | ENSBTAG00000016290 | protein_coding |
| *SLC4A4* | 6 | 86449877 | 86813494 | ENSBTAG00000002348 | protein_coding |
| *GC* | 6 | 86963822 | 87007062 | ENSBTAG00000013718 | protein_coding |
| *COX18* | 6 | 88173347 | 88184845 | ENSBTAG00000005394 | protein_coding |
| *ADAMTS3* | 6 | 87426966 | 87707488 | ENSBTAG00000006507 | protein_coding |
|  | 6 | 85829450 | 85830830 | ENSBTAG00000007816 | protein_coding |
| *UTP3* | 6 | 86071006 | 86074371 | ENSBTAG00000009310 | protein_coding |
|  | 6 | 85587463 | 85590100 | ENSBTAG00000069370 | lncRNA |
|  | 6 | 85624624 | 85632447 | ENSBTAG00000070501 | lncRNA |
|  | 6 | 85567225 | 85591611 | ENSBTAG00000061427 | lncRNA |
|  | 6 | 86376751 | 86446957 | ENSBTAG00000057428 | lncRNA |
| *EDNRB* | 12 | 53038377 | 53068132 | ENSBTAG00000005299 | protein_coding |
| *POU4F1* | 12 | 53744577 | 53746732 | ENSBTAG00000051184 | protein_coding |
| *OBI1* | 12 | 53756338 | 53802438 | ENSBTAG00000000869 | protein_coding |
| *SLAIN1* | 12 | 52840174 | 52892769 | ENSBTAG00000003511 | protein_coding |
|  | 12 | 53741809 | 53743551 | ENSBTAG00000059456 | lncRNA |
| *U6* | 12 | 53853456 | 53853562 | ENSBTAG00000043485 | snRNA |
| *bta-mir-2284s* | 12 | 52903108 | 52903175 | ENSBTAG00000044565 | miRNA |
| *U6* | 12 | 51078999 | 51079105 | ENSBTAG00000078406 | snRNA |

**Table 5.** Gene Ontology terms for the genes annotated for depigmentation trait after conditional GWAS

| Functional Terms | source | Description of function | genes |
| --- | --- | --- | --- |
| GO:0051145 | GO:BP | smooth muscle cell differentiation | ENSBTAG00000002699,ENSBTAG00000014601,ENSBTAG00000004912,ENSBTAG00000010273,ENSBTAG00000005299 |
| GO:0051923 | GO:BP | sulfation | ENSBTAG00000001249,ENSBTAG00000038214,ENSBTAG00000011952 |
| GO:1901135 | GO:BP | carbohydrate derivative metabolic process | ENSBTAG00000001249,ENSBTAG00000011952,ENSBTAG00000014913,ENSBTAG00000001269,ENSBTAG00000002699,ENSBTAG00000010571,ENSBTAG00000021347,ENSBTAG00000005078,ENSBTAG00000000287,ENSBTAG00000012397,ENSBTAG00000002348,ENSBTAG00000007816,ENSBTAG00000005299 |
| GO:0046164 | GO:BP | alcohol catabolic process | ENSBTAG00000001249,ENSBTAG00000011952,ENSBTAG00000014913 |
| GO:0042692 | GO:BP | muscle cell differentiation | ENSBTAG00000002699,ENSBTAG00000014601,ENSBTAG00000002333,ENSBTAG00000007173,ENSBTAG00000005078,ENSBTAG00000004912,ENSBTAG00000010273,ENSBTAG00000005299 |
| GO:0007214 | GO:BP | gamma-aminobutyric acid signaling pathway | ENSBTAG00000016645,ENSBTAG00000017837,ENSBTAG00000011817 |
| GO:1902710 | GO:CC | GABA receptor complex | ENSBTAG00000016645,ENSBTAG00000017837,ENSBTAG00000011817 |
| GO:1902711 | GO:CC | GABA-A receptor complex | ENSBTAG00000016645,ENSBTAG00000017837,ENSBTAG00000011817 |
| GO:0004713 | GO:MF | protein tyrosine kinase activity | ENSBTAG00000009438,ENSBTAG00000000782,ENSBTAG00000005055,ENSBTAG00000005062,ENSBTAG00000002699,ENSBTAG00000007173,ENSBTAG00000004052,ENSBTAG00000010273,ENSBTAG00000018134 |
| GO:0004714 | GO:MF | transmembrane receptor protein tyrosine kinase activity | ENSBTAG00000009438,ENSBTAG00000000782,ENSBTAG00000002699,ENSBTAG00000007173,ENSBTAG00000004052,ENSBTAG00000010273,ENSBTAG00000018134 |
| GO:0019199 | GO:MF | transmembrane receptor protein kinase activity | ENSBTAG00000009438,ENSBTAG00000000782,ENSBTAG00000002699,ENSBTAG00000007173,ENSBTAG00000004052,ENSBTAG00000010273,ENSBTAG00000018134 |
| GO:0004062 | GO:MF | aryl sulfotransferase activity | ENSBTAG00000001249,ENSBTAG00000038214,ENSBTAG00000011952 |
| GO:0030297 | GO:MF | transmembrane receptor protein tyrosine kinase activator activity | ENSBTAG00000004052,ENSBTAG00000010273,ENSBTAG00000018134 |
| GO:0038085 | GO:MF | vascular endothelial growth factor binding | ENSBTAG00000000782,ENSBTAG00000007173 |
| GO:0030296 | GO:MF | protein tyrosine kinase activator activity | ENSBTAG00000004052,ENSBTAG00000010273,ENSBTAG00000018134 |
| GO:0005021 | GO:MF | vascular endothelial growth factor receptor activity | ENSBTAG00000000782,ENSBTAG00000007173 |
| GO:0016740 | GO:MF | transferase activity | ENSBTAG00000009438,ENSBTAG00000059934,ENSBTAG00000001249,ENSBTAG00000038214,ENSBTAG00000011952,ENSBTAG00000000782,ENSBTAG00000005055,ENSBTAG00000005062,ENSBTAG00000044044,ENSBTAG00000019366,ENSBTAG00000002699,ENSBTAG00000010571,ENSBTAG00000007173,ENSBTAG00000020658,ENSBTAG00000004052,ENSBTAG00000010273,ENSBTAG00000018134,ENSBTAG00000012397,ENSBTAG00000016290,ENSBTAG00000007816,ENSBTAG00000000869 |
| GO:0016773 | GO:MF | phosphotransferase activity, alcohol group as acceptor | ENSBTAG00000009438,ENSBTAG00000000782,ENSBTAG00000005055,ENSBTAG00000005062,ENSBTAG00000002699,ENSBTAG00000007173,ENSBTAG00000004052,ENSBTAG00000010273,ENSBTAG00000018134,ENSBTAG00000012397,ENSBTAG00000016290 |
| GO:0004672 | GO:MF | protein kinase activity | ENSBTAG00000009438,ENSBTAG00000000782,ENSBTAG00000005055,ENSBTAG00000005062,ENSBTAG00000002699,ENSBTAG00000007173,ENSBTAG00000004052,ENSBTAG00000010273,ENSBTAG00000018134,ENSBTAG00000016290 |
| GO:0016301 | GO:MF | kinase activity | ENSBTAG00000009438,ENSBTAG00000000782,ENSBTAG00000005055,ENSBTAG00000005062,ENSBTAG00000002699,ENSBTAG00000007173,ENSBTAG00000004052,ENSBTAG00000010273,ENSBTAG00000018134,ENSBTAG00000012397,ENSBTAG00000016290 |
| GO:0016772 | GO:MF | transferase activity, transferring phosphorus-containing groups | ENSBTAG00000009438,ENSBTAG00000000782,ENSBTAG00000005055,ENSBTAG00000005062,ENSBTAG00000019366,ENSBTAG00000002699,ENSBTAG00000007173,ENSBTAG00000004052,ENSBTAG00000010273,ENSBTAG00000018134,ENSBTAG00000012397,ENSBTAG00000016290 |
| GO:0030295 | GO:MF | protein kinase activator activity | ENSBTAG00000004052,ENSBTAG00000010273,ENSBTAG00000018134,ENSBTAG00000016290 |
| GO:0022851 | GO:MF | GABA-gated chloride ion channel activity | ENSBTAG00000017837,ENSBTAG00000011817 |
| GO:0019209 | GO:MF | kinase activator activity | ENSBTAG00000004052,ENSBTAG00000010273,ENSBTAG00000018134,ENSBTAG00000016290 |
| GO:0008146 | GO:MF | sulfotransferase activity | ENSBTAG00000001249,ENSBTAG00000038214,ENSBTAG00000011952 |
| HP:0000705 | HP | Amelogenesis imperfecta | ENSBTAG00000001269,ENSBTAG00000002928,ENSBTAG00000004793,ENSBTAG00000010346 |
| HP:0001901 | HP | Polycythemia | ENSBTAG00000054580,ENSBTAG00000011789,ENSBTAG00000068505,ENSBTAG00000005299 |
| KEGG:00140 | KEGG | Steroid hormone biosynthesis | ENSBTAG00000064079,ENSBTAG00000039991,ENSBTAG00000059934,ENSBTAG00000011952,ENSBTAG00000058539,ENSBTAG00000053282,ENSBTAG00000014913 |
| KEGG:00053 | KEGG | Ascorbate and aldarate metabolism | ENSBTAG00000064079,ENSBTAG00000039991,ENSBTAG00000059934,ENSBTAG00000058539,ENSBTAG00000053282 |
| KEGG:00040 | KEGG | Pentose and glucuronate interconversions | ENSBTAG00000064079,ENSBTAG00000039991,ENSBTAG00000059934,ENSBTAG00000058539,ENSBTAG00000053282 |
| KEGG:00860 | KEGG | Porphyrin metabolism | ENSBTAG00000064079,ENSBTAG00000039991,ENSBTAG00000059934,ENSBTAG00000058539,ENSBTAG00000053282 |
| KEGG:04976 | KEGG | Bile secretion | ENSBTAG00000064079,ENSBTAG00000039991,ENSBTAG00000059934,ENSBTAG00000058539,ENSBTAG00000053282,ENSBTAG00000002348 |
| KEGG:00982 | KEGG | Drug metabolism - cytochrome P450 | ENSBTAG00000064079,ENSBTAG00000039991,ENSBTAG00000059934,ENSBTAG00000058539,ENSBTAG00000053282 |
| KEGG:05204 | KEGG | Chemical carcinogenesis - DNA adducts | ENSBTAG00000064079,ENSBTAG00000039991,ENSBTAG00000059934,ENSBTAG00000058539,ENSBTAG00000053282 |
| KEGG:00980 | KEGG | Metabolism of xenobiotics by cytochrome P450 | ENSBTAG00000064079,ENSBTAG00000039991,ENSBTAG00000059934,ENSBTAG00000058539,ENSBTAG00000053282 |
| KEGG:00830 | KEGG | Retinol metabolism | ENSBTAG00000064079,ENSBTAG00000039991,ENSBTAG00000059934,ENSBTAG00000058539,ENSBTAG00000053282 |
| KEGG:00983 | KEGG | Drug metabolism - other enzymes | ENSBTAG00000064079,ENSBTAG00000039991,ENSBTAG00000059934,ENSBTAG00000058539,ENSBTAG00000053282 |
| KEGG:05033 | KEGG | Nicotine addiction | ENSBTAG00000016645,ENSBTAG00000017837,ENSBTAG00000012296,ENSBTAG00000011817 |
| KEGG:01240 | KEGG | Biosynthesis of cofactors | ENSBTAG00000064079,ENSBTAG00000039991,ENSBTAG00000059934,ENSBTAG00000058539,ENSBTAG00000053282 |
| KEGG:04727 | KEGG | GABAergic synapse | ENSBTAG00000016645,ENSBTAG00000017837,ENSBTAG00000012296,ENSBTAG00000011817 |
| KEGG:05032 | KEGG | Morphine addiction | ENSBTAG00000016645,ENSBTAG00000017837,ENSBTAG00000012296,ENSBTAG00000011817 |
| KEGG:05207 | KEGG | Chemical carcinogenesis - receptor activation | ENSBTAG00000064079,ENSBTAG00000039991,ENSBTAG00000059934,ENSBTAG00000058539,ENSBTAG00000053282 |
| KEGG:04723 | KEGG | Retrograde endocannabinoid signaling | ENSBTAG00000016645,ENSBTAG00000017837,ENSBTAG00000012296,ENSBTAG00000011817 |
| REAC:R-BTA-180336 | REAC | SHC1 events in EGFR signaling | ENSBTAG00000004052,ENSBTAG00000010273,ENSBTAG00000018134 |
| REAC:R-BTA-9753281 | REAC | Paracetamol ADME | ENSBTAG00000064079,ENSBTAG00000039991,ENSBTAG00000059934,ENSBTAG00000011952 |
| REAC:R-BTA-179812 | REAC | GRB2 events in EGFR signaling | ENSBTAG00000004052,ENSBTAG00000010273,ENSBTAG00000018134 |
| REAC:R-BTA-212718 | REAC | EGFR interacts with phospholipase C-gamma | ENSBTAG00000004052,ENSBTAG00000010273,ENSBTAG00000018134 |
| REAC:R-BTA-180292 | REAC | GAB1 signalosome | ENSBTAG00000004052,ENSBTAG00000010273,ENSBTAG00000018134 |
| REAC:R-BTA-9757110 | REAC | Prednisone ADME | ENSBTAG00000064079,ENSBTAG00000039991,ENSBTAG00000059934 |
| REAC:R-BTA-6811558 | REAC | PI5P, PP2A and IER3 Regulate PI3K/AKT Signaling | ENSBTAG00000002699,ENSBTAG00000007173,ENSBTAG00000004052,ENSBTAG00000010273,ENSBTAG00000018134 |
| REAC:R-BTA-199418 | REAC | Negative regulation of the PI3K/AKT network | ENSBTAG00000002699,ENSBTAG00000007173,ENSBTAG00000004052,ENSBTAG00000010273,ENSBTAG00000018134 |
| REAC:R-BTA-182971 | REAC | EGFR downregulation | ENSBTAG00000004052,ENSBTAG00000010273,ENSBTAG00000018134 |
| REAC:R-BTA-5223345 | REAC | Miscellaneous transport and binding events | ENSBTAG00000007695,ENSBTAG00000009423,ENSBTAG00000039787 |
| REAC:R-BTA-9748784 | REAC | Drug ADME | ENSBTAG00000064079,ENSBTAG00000039991,ENSBTAG00000059934,ENSBTAG00000011952 |
| REAC:R-BTA-177929 | REAC | Signaling by EGFR | ENSBTAG00000004052,ENSBTAG00000010273,ENSBTAG00000018134 |
| REAC:R-BTA-9006934 | REAC | Signaling by Receptor Tyrosine Kinases | ENSBTAG00000000782,ENSBTAG00000005062,ENSBTAG00000019366,ENSBTAG00000002699,ENSBTAG00000007173,ENSBTAG00000004052,ENSBTAG00000010273,ENSBTAG00000018134 |
| WP:WP3127 | WP | Cardiac progenitor differentiation | ENSBTAG00000000782,ENSBTAG00000002699,ENSBTAG00000007173 |
| WP:WP3234 | WP | Sulfation biotransformation reaction | ENSBTAG00000001249,ENSBTAG00000011952 |


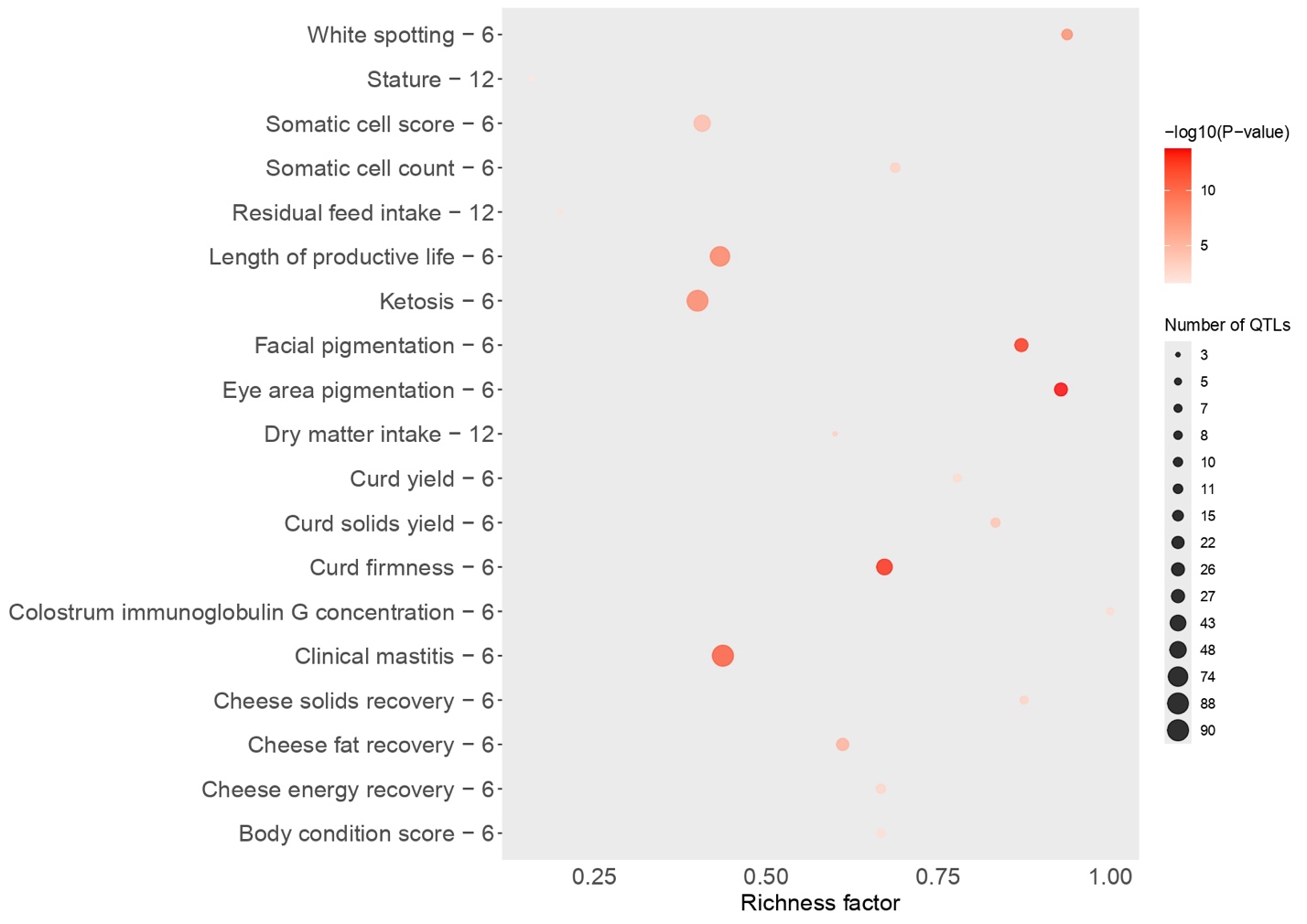


**Fig 1.** Quantitative trait loci (QTL) enrichment for the significant regions for the depigmentation trait after conditional GWAS.
